# Impact of Prenatal Stress on Neuroendocrine Programming

**DOI:** 10.1100/tsw.2007.204

**Published:** 2007-09-01

**Authors:** Odile Viltart, Christel C. A. Vanbesien-Mailliot

**Affiliations:** ^1^ NeuroImmunoEndocrinology Laboratory, Pasteur Institute of Lille, F-59019 Lille, France; ^2^ EA2683 MENRT, Clinics and Physiopathology of Parkinson's Disease, Lille Medical School, University of Lille 2, F-59045 Lille, France; ^3^ Department of Adaptive Neuroscience and Physiology, UPRES EA 4052, University of Lille 1, F-59655 Villeneuve d'Ascq, France

**Keywords:** adaptation, central nervous system, developmental windows, environment, evolution, feto-placental unit, gestational stress, glucocorticoid, growth, homeostasis, hypothalamo-pituitary-adrenal axis, immune function, imprinting, maternal care, metabolic syndrome, mood disorders, neuroimmunoendocrinology, obesity, plasticity, programming, sexual behavior, stress, sympathetic nervous system

## Abstract

Since life emerged on the Earth, the development of efficient strategies to cope with sudden and/or permanent changes of the environment has been virtually the unique goal pursued by every organism in order to ensure its survival and thus perpetuate the species. In this view, evolution has selected tightly regulated processes aimed at maintaining stability among internal parameters despite external changes, a process termed homeostasis. Such an internal equilibrium relies quite heavily on three interrelated physiological systems: the nervous, immune, and endocrine systems, which function as a permanently activated watching network, communicating by the mean of specialized molecules: neurotransmitters, cytokines, and hormones or neurohormones. Potential threats to homeostasis might occur as early as during *in utero* life, potentially leaving a lasting mark on the developing organism. Indeed, environmental factors exert early-life influences on the structural and functional development of individuals, giving rise to changes that can persist throughout life. This organizational phenomenon, encompassing prenatal environmental events, altered fetal growth, and development of long-term pathophysiology, has been named early-life programming. Over the past decade, increased scientific activities have been devoted to deciphering the obvious link between states of maternal stress and the behavioral, cognitive, emotional, and physiological reactivity of the progeny. This growing interest has been driven by the discovery of a tight relationship between prenatal stress and development of short- and long-term health disorders. Among factors susceptible of contributing to such a deleterious programming, nutrients and hormones, especially steroid hormones, are considered as powerful mediators of the fetal organization since they readily cross the placental barrier. In particular, variations in circulating maternal glucocorticoids are known to impact this programming strongly, notably when hormonal surges occur during sensitive periods of development, so-called developmental windows of vulnerability. Stressful events occurring during the perinatal period may impinge on various aspects of the neuroendocrine programming, subsequently amending the offspring's growth, metabolism, sexual maturation, stress responses, and immune system. Such prenatal stress-induced modifications of the phenotypic plasticity of the progeny might ultimately result in the development of long-term diseases, from metabolic syndromes to psychiatric disorders. Yet, we would like to consider the outcome of this neuroendocrine programming from an evolutionary perspective. Early stressful events during gestation might indeed shape internal parameters of the developing organisms in order to adapt the progeny to its everyday environment and thus contribute to an increased reproductive success, or fitness, of the species. Moreover, parental care, adoption, or enriched environments after birth have been shown to reverse negative long-term consequences of a disturbed gestational environment. In this view, considering the higher potential for neonatal plasticity within the brain in human beings as compared to other species, long-term consequences of prenatal stress might not be as inexorable as suggested in animal-based studies published to date.

## INTRODUCTION

Since the early origins of life on Earth, the survival of living organisms, from the simplest protozoan to the most complicated metazoan, has always depended on their ability to cope with environmental vicissitudes. In order to maintain their internal condition within tolerable limits in face of the many changes of their environment, a process called “homeostasis”, cells or individuals have developed ingenious adaptive mechanisms that allow their immediate survival and thus the survival of their species. Indeed, in a permanent dynamic interaction with the environment, metazoans continuously adjust their physiology to these ever-changing conditions. Whatever the complexity of the organism considered, such constant modifications require a high level of plasticity achieved by the means of multiple dynamic equilibrium adjustments that are controlled by tightly interrelated regulation mechanisms. In this view, the survival of individuals relies on a seemingly endless repertoire of neural, endocrine, and immunological responses, enabling them to cope with physical, chemical, and biological disturbances. These physiological and/or behavioral adaptive changes are the expression of a well-balanced transitional state developed by the organism before returning to homeostasis, a transitional state named “allostasis”. This term, literally meaning “*maintaining stability through change*”, was first introduced by Sterling and Eyer[313] to describe physiological changes occurring in organisms facing physical, physiological, or psychological stressors. The various consecutive changes/adaptations result from a narrow cross-talk between genes and the intrinsic or extrinsic environment. Indeed, genes contain specific information on which the environment can exert a pressure (that can be assimilated to the pressure of selection) in order to allow the emergence of a particular phenotype or another[312]. This so-called phenotypic plasticity is observed throughout phylogenesis[345] and arose through hundreds of millions of years of vertebrate evolution. Indeed, every vertebrate responds to stressful situations by the activation of the sympathetic branch of its autonomic nervous system (a response initially coined “*the fight-or-flight*” response or acute stress response by Cannon[39]) followed by secretions of hormones by its hypothalamo-pituitary-adrenal (HPA) axis[296,297]. Among the secreted hormones, the corticotrophin-releasing hormone (CRH), one of the major hypothalamic neurohormones, controls the release of corticosteroids by the adrenal cortex *via* the activation of pituitary adrenocorticotropic cells[274] (Fig. 1). This CRH system is also found in invertebrates and probably evolved from ancestral species existing before the Precambrian explosion, as recently shown by Lovejoy and Jahan[198]. Thus, such survival mechanisms have been selected through complex evolutive processes that emerged in millions of years to allow a mobilization of the individuals’ neuroendocrine system, in order to raise their basal energy level so that they can accurately face stressful situations.

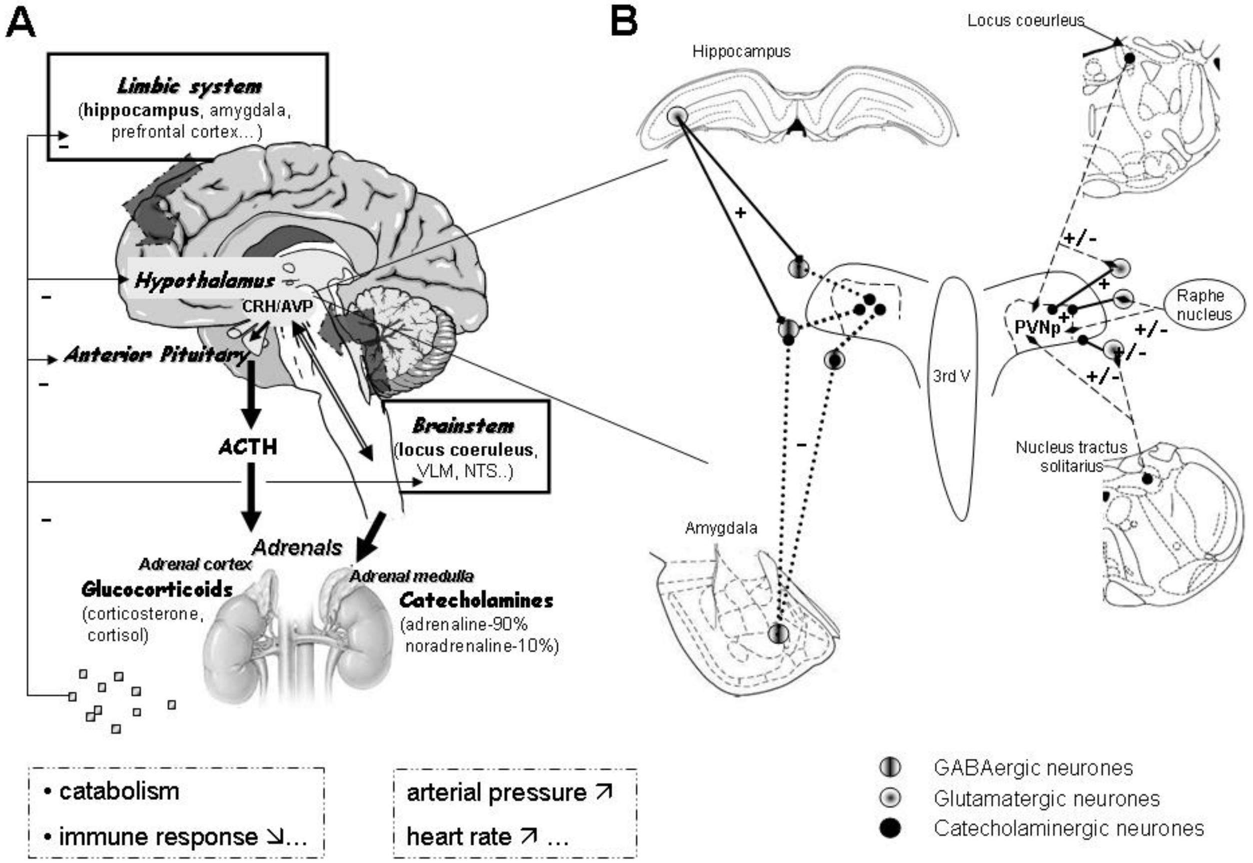

**FIGURE 1**. Functional anatomy of the stress response. (A) Stressful situations activate various brain structures, including the limbic system and brainstem, which results in the stimulation of the hypothalamic paraventricular nucleus (PVN). PVN neurons release corticotrophin-releasing hormone (CRH)/arginine-vasopressin (AVP) that induce adrenocorticotropin hormone (ACTH) secretion from the anterior hypophysis. In turn, ACTH acts on the adrenal cortex and triggers glucocorticoid (GC) liberation. These steroid hormones target various organs to mobilize energy and control the brain *via* a negative feedback. In parallel, sympathetic brain areas located in particular hypothalamic (PVN) and brainstem nuclei (nucleus tractus solitarius, etc.) control the catecholamine release from adrenal medulla chromaffin cells. Adrenaline and noradrenaline target organs to induce an adapted physiological response to acute stress. (B) Detail of the fine regulation of PVN. The parvocellular part of PVN is controlled by a neuronal microenvironment composed of GABAergic and glutamatergic neurons. These interneurons as well as CRH neurons are the targets of regulatory inputs coming from hippocampus (glutamatergic afferences), amygdala (GABAergic afferences), locus coeruleus and nucleus tractus solitarius (noradrenergic afferences), and raphe nucleus (serotoninergic afferences). Indeed, the hippocampus exerts a strong tonic regulatory modulation *via* activation of mineralocorticoid receptors (MR) that bind plasma GC with a high affinity. These receptors are mainly occupied in basal situation when the plasma GC levels are low (Reul and de Kloet, 1985[269]). In stressful situations, the rise in GC affects both hippocampal MR and GC receptors (GR), and thus inhibits the activity of PVN cells. The hippocampal GR are involved in the phasic inhibition of PVN; there are parts of the feedback control of the HPA axis. PVN is also targeted by noradrenergic inputs arising from the locus coeruleus and nucleus tractus solitarius; these noradrenergic fibers directly or indirectly modulate the activity of CRF/AVP cells. In fact, the PVN is surrounded by a "microenvironment" composed by GABAergic and glutamatergic interneurons. Among other brain areas that are known to modulate the PVN in stress, the amygdala and the prefrontal cortex project in or around the PVN, thus exerting inhibitory or excitatory stimulations on PVN. Scheme adapted from Herman et al. (2002) (*Pharmacol. Biochem. Behav.*, **71**, 457-468).

In the context of the theory of life history, another kind of plasticity has to be emphasized. While environmental influences are often considered to begin after birth, many studies have emphasized the importance of the prenatal *in utero* environment[64,72,73,74,142,235]. In this view, individuals are also exposed to the developmental plasticity, a process defined by Horton[142] as "*a genetically based program that can be modified in response to changing environmental conditions to shape the unique characteristics of each individual*." In fact, these environmental conditions can produce different outcomes on the development of the organism. Such conditions can indeed be either advantageous and promote an optimal development, or deleterious and alter the development[26]. Therefore, if the environmental context during gestation is perceived by the dam as stressful or hostile (in the presence of a potential predator, for example), the resulting activation of the maternal HPA axis may alter essential developmental processes of the fetus. Depending on the duration of the gestational stress (acute or chronic), stress-induced maternal hormonal secretions might program deleterious short- and/or long-term consequences for the health of the offspring. Thus, early disturbances (both pre- and postnatal) occurring at specific sensitive developmental periods may extend their influences throughout the life of animals and human beings.

To investigate the characteristics of these sensitive periods, the use of animal models remains essential. Indeed, various experimental models have provided evidences that support a causal role of perinatal stress on the developing organism in the occurrence of long-term physiological and/or psychological disturbances. For instance, undernutrition, psychological stress, or hypoxia during gestation have been shown to impair the physiological development of the offspring (Table 1). However, the consequences of these changes on developmental pathways strongly depend on the timing at which such environmental events take place. Indeed, discrepant results reported in the literature are often related to differences between early stress (from the first third of the gestation on) and late stress (lasting during the last third of gestation only), or to the gestational paradigm as well as to the animal species used (Table 1). Nevertheless, whatever the species considered and the gestational stress paradigm applied, the major consequence observed in prenatally stressed newborns is a lower birth weight as compared to pups generated from unstressed dams. In addition, adverse effects of prenatal stress (PS) can not only alter the brain morphology of the offspring, the time course of normal aging, and the longevity of individual, but it can also affect neuroendocrine systems, thus leading to a reduced growth rate, an altered sexual differentiation, an inappropriate stress response, and immune dysfunctions. These physiological and behavioral alterations are thought to be programmed during fetal development, as initially suggested by studies in human beings[17,199]. Even if the precise mechanisms of prenatal programming still remain unclear, it is now widely accepted that prenatal influences on the offspring are mediated by the maternal response to stress, and more especially through maternal stress hormones secreted by the pituitary gland (adrenocorticotropic hormone or ACTH) and adrenals (namely glucocorticoids, epinephrine, and norepinephrine). In fact, glucocorticoids (GC) are known to act on different organs *via* mineralocorticoid or GC receptors (MR and GR or type I and II receptors)[269], therefore exerting a crucial effect on their development and function. Thus, a disturbed gestation can modify the setup and the organizational patterns of the developing individual's physiological circuits. Thereby, long-lasting prenatal alterations are suspected of increasing the risk for an individual to develop diseases at adulthood, such as type II diabetes, obesity, hypertension, as well as anxiety-like behaviors. In this view, most PS consequences described in humans have been replicated in various animal models[223].

From an evolutionary perspective, PS can be conceived as an adaptive response aimed at favoring the immediate survival of the fetus in a disturbed gestational environment, allowing the species perennity. However, beyond this immediate protective role, PS long-term consequences on the individuals' physiology might generate a maladapted phenotype. Many hypotheses are proposed to pinpoint the evolutionary significance of the responses observed in PS progenies[200]. On one hand, the fetal programming hypothesis suggests an alteration of the fetal genome that would lead to permanent effects on various physiological processes through early changes in nutrients and hormonal conditions[199]. On the other hand, Hales and Barker[126] proposed the "thrifty phenotype" hypothesis according to which the fetal growth rate might set nutritional expectancies to enable an adequate nutritional behavior later in the organism's life[168]. In a recent study, Bateson and coworkers further stated the crucial role of the fetal physiological responsiveness to maternal condition in the preparation and adaptation for facing future challenging environments[21]. However, the current human way of life has considerably changed compared to earlier times in human evolutive history (from a nutritional point of view, for example); such a fetal programming may thus lead to damaging consequences on health. In other words, adaptive processes that have been selected through evolution for enabling primitive individuals to face stressful environments several millions of years ago might not be accurate anymore, and thus could turn out to be disadvantageous in modern life.

**TABLE 1**
**Nonexhaustive List of Different Experimental Paradigms of PS Widely Used in Several Species for Modeling PS-Induced Alterations of Human Beings**

| Animal Species | Prenatal Stress Procedure | Timing | Ref. |
|---|---|---|---|
| Rhesus monkey | Dexamethasone treatment (0.125 mg/kg BW) | GD145–146 | [53] |
| | Disturbed gestation* | _Early_: GD50–92 or _Late_: GD105–147 | |
| | Daily ACTH injection (1 USP unit/kg BW) | GD120–133 | [52] |
| Lamb | Endotoxin IA injections or betamethasone IM injections or a combination of both | GD108–110 | [152] |
| Pig | Daily physical restraint with a nose sling for 5 min | Week 12–16 | [330] |
| Rat (Sprague Dawley) | Daily restraint in a transparent plastic cylinder under bright light for 45 min three times per day | GD15–21; GD10–21 | [202,342] |
| | Daily hanging for 2 h | GD14–21 | [192] |
| | Unpredictible noise and flashing lights three times per week | GD1–21 | [99,156] |
| | Daily exposure to an 85- or 90-dB fire alarm bell (30 times for 1 h) | GD15-21 | [309] |
| | _Environmental stress_: 15 unsignaled inescapable electric foot shock (30 min/day) | GD15-21 | [308] |
| | _Psychological stress_: placement of the pregnant dam in the nonelectrified section of the shock apparatus in order to see, hear, and smell a nonpregnant partner submitted to environmental stress | GD15-21 | |
| | Injection of 20 µg CRH (l-41 rat/human) in a volume of 0.1 ml saline, subcutaneously into the nape of the neck. Two injections of CRH, with an interval between injections that was never less than 6 h | GD14–21 | [371] |
| | Pregnant females were placed in a normobaric Plexiglas chamber supplied with a gas mixture consisting of 10% $O_2$/90% $N_2$ (temperature within the chamber:26°± 1°C) | GD5–20 | [281] |
| | One day before delivery, pregnant females replaced in the normoxic room at 21% $O_2$ | | |
| Rat (Wistar) | Injection i.p. of LPS (_Escherichia coli_; 30 µg/kg) or human red blood cells ($5 \times 10^8$ in 300 µl NaCl) | At GD10 | [270] |
| | IP injections of dexamethasone (1.2 mg/kg BW), twice a day | GD11 and 18 | [12] |
| | IP injections of morphine sulfate (10 mg/kg BW) twice a day or with physiological saline (0.9% NaCl). | GD11-18 | [184,343] |
| | Control diet (180 g casein/kg) _vs._ low protein diet (90 g casein/kg); diets balanced in energy (28 MJ/kg diet), fat, fiber, and micronutrient content | GD0 to term | [171,228] |
| | Intraperitoneal injections of human IL-6 (9 µg/kg dissolved in saline phosphate buffer) | _Early IL-6 exposure_: injection at GD8, 10, and 12<br>_Late IL-6 exposure_: injection at GD16, 18, and 20 | [286] |
| Rat (DA/HAN) | _Acute stress_ : pregnant female and cat were put together for two periods of 15 min spaced by a 15-min interval (between 10 a.m. and 12 p.m.) | At GD10 or 14 | [46,195,196] |
| | _Repeated stress_: pregnant female and cat were put together for ten periods of 10 min spaced by a 30-min interval (between 9 a.m. and 5 p.m.) | | |
| Rat (Long Ewans) | Daily restraint stress under bright light and increased temperature (room temperature at 38°C) for 45 min three times per day | GD14–21 | [163] |

* Disturbed gestation corresponds to the daily relocation of the pregnant female in a darkened room for 10 min between 2:30 and 4:00 pm associated with an acoustic startle protocol (1 sec, three times randomly during the 10 min of relocation).

BW: body weight; GD: gestational day; IA: intrauterine; IM: intramuscular; IP: intraperitoneal.

Considering the importance of the fetal responsiveness to maternal stress in the potential emergence of diseases of the adult[17], the present review aims to delineate neuroendocrine changes observed in fetuses that have experienced chronic gestational stress, as well as their consequences throughout life. We will first focus the reader's attention onto the PS-evoked growth retardation. The second section will examine PS consequences on the development of sexual abilities from both a behavioral and a hormonal point of view, as well as its impact on sexual brain differentiation. Our third point is intended to clarify the relationships between these dysfunctions and the programming of the HPA axis. In the fourth section, we will question the potential impact of a disturbed gestational environment on the immune system of the fetal, neonatal, juvenile, and adult offspring. We will also consider several proposed mechanisms that might underlie the potential alterations of immunity. Lastly, since PS is considered as a vulnerability factor to develop short- and long-term disorders, we will review various PS-induced diseases outlined in animal models and human beings, as well as the potential reversion of these adverse outcomes.

## PRENATAL STRESS REDUCES GROWTH DEVELOPMENT

Life expectancy is closely related to intrauterine events that control the fetal development and thus the birth weight. The common feature of any gestational stress is a reduction of the birth weight as a result of intrauterine growth retardation (IUGR), which is associated with a significant neonatal and childhood morbidity. In developing countries, an estimated 30 million infants are born each year with IUGR; 75% of them in Asia and 20% in Africa. In our industrialized countries, children born with abnormally low birth weights are at increased risk for later overweight and obesity[183,233]. Indeed, the rapid weight gain or catch-up growth, referring to an accelerated growth beyond the normal rate for the age considered[103], frequently noticed after birth when food is available, may alter energy metabolism and/or feeding behaviors, predisposing individuals to overweight or obesity[238].

### Role of Nutrients in Growth

Placental alterations and gestational stress are the main causes of IUGR[91,302]. In mammals, the placenta is a major determinant of intrauterine growth. It controls all mother-to-fetus transfers, thus allowing the supply of the developing organism with nutrients and/or maternal hormones that cross this barrier by diffusion and transporters (Fig. 2)[301]. Both external and endogenous PS-evoked factors affect the integrity of the feto-placental unit. On the one hand, external factors that reduce uterine blood flow restrict the fetal nutrient and/or oxygen supply, and may thus initiate a fetal stress response. For example, maternal anxiety in the third trimester in human beings is associated with an increased uterine artery resistance index that results in alterations of the fetal development and therefore might account for the low birth weight[323]. On the other hand, the determinant role of maternal nutrition in controlling fetal growth has been underlined in numerous studies. Hence, both PS animal models and human cohort studies have demonstrated the impact of undernutrition during pregnancy onto fetal growth retardation[223]. Most animal studies were performed on rodents, although other species have been used as well (Table 1). A primary nutrient for both the mother and the developing fetus is glucose, which crosses the placental barrier through facilitated transporters following a maternal-to-fetal concentration gradient[150]. These transporters, GLUT1, GLUT3, and GLUT4, are found both in human and rodent placentas[167]. Considering the fundamental role of these transporters in providing the fetus with an essential nutrient for its growth, one can easily foresee the detrimental consequences PS could have on their function (Fig. 2). In fact, we found a strong decrease of GLUT1 protein levels in PS rat feto-placental unit paralleled with fetal hypoglycemia, whereas GLUT3 and GLUT4 were augmented[204]. Since GLUT1 is a rate-limiting transporter for glucose from the mother to the fetal organism, this low glycemia could be a direct consequence of altered GLUT expression[22]. These effects could be related to the reduced maternal food intake during pregnancy[160,204,352]. However, with the exception of deliberate procedures of maternal

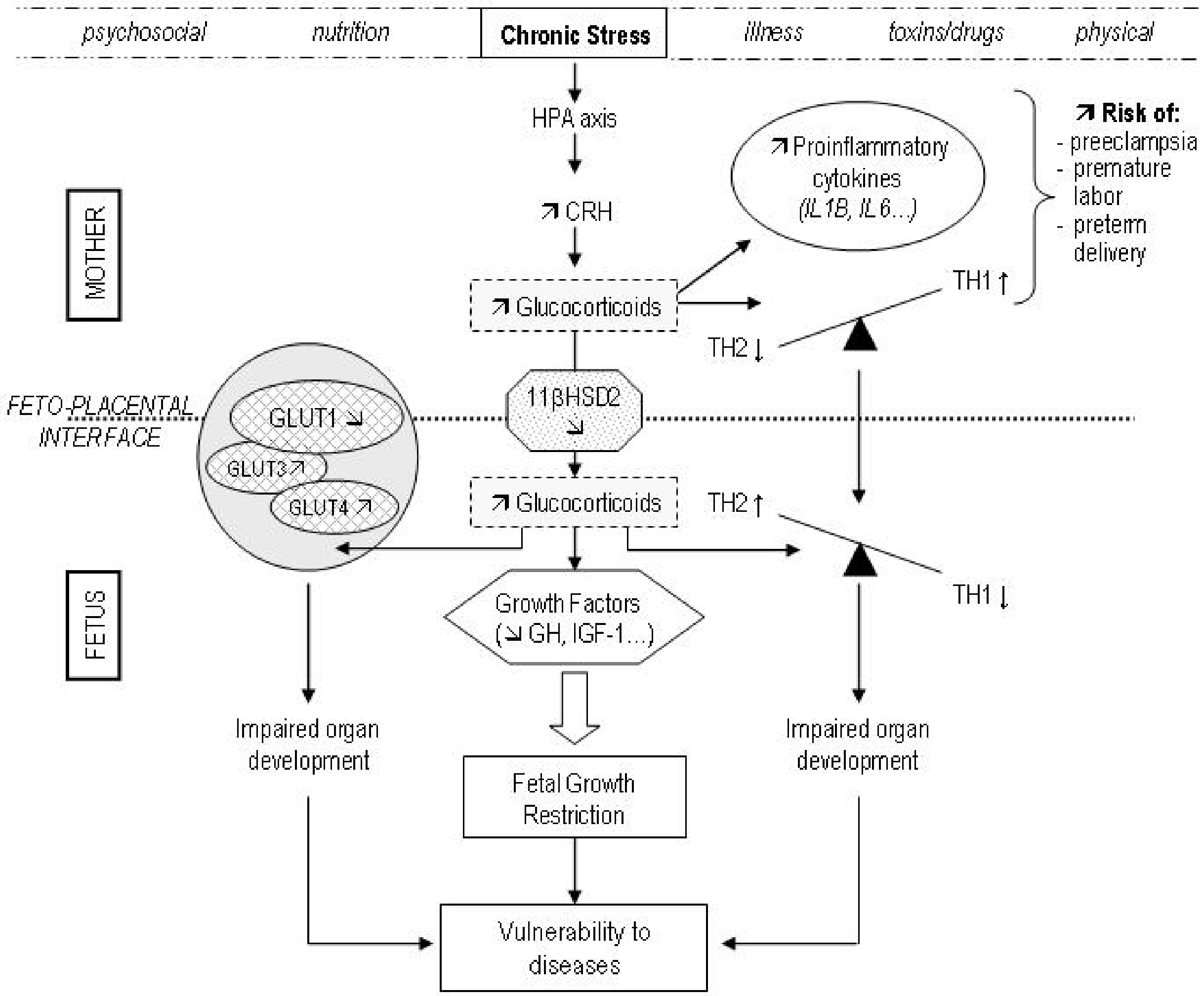

**FIGURE 2.** Consequences of chronic gestational stress at the feto-placental interface. Chronic stress during pregnancy, whether physical, psychosocial, nutritional, immune, or toxic, activates the maternal HPA axis, notably by increasing the production of corticotrophin-releasing hormone (CRH) and glucocorticoid (GC). Such changes within the maternal organism have been shown to increase the production of proinflammatory cytokines, such as interleukin (IL) 1B and IL-6[62], thereby shifting the T helper (Th) 1/Th2 balance towards a Th-2 proinflammatory profile, which has been associated with the occurrence of preeclampsia and premature labor/preterm delivery[254]. The chronic gestational stress alters fetal development–altered transfer of nutrients and/or hormones through the placenta. In this case, the glucose transport is detrimental, leading to impaired organ development. The excess GC in the feto-placental unit derives from the elevated maternal GC levels. The transfer of maternal GC is facilitated by the decrease in both activity and expression of the 11β-HSD2, a placental enzyme that functions to protect the fetus from an excessive transfer of maternal GC. All of these factors intervene directly or indirectly in the development of intrauterine growth retardation. Gestational stress may thus alter the neuroendocrine programming, leading to low birth weight progeny that present an increased vulnerability to develop long-term metabolic and psychiatric diseases. 11β-HSD2: 11 β -hydroxysteroid dehydrogenase type 2; GH: growth hormone; GLUT: glucose transporter; HPA: hypothalamic-pituitary-adrenal axis; IGF-1: insulin growth factor type 1; TH: T helper lymphocyte subset.

undernutrition (Table 1), most gestational paradigms mimicking psychological stress do not provoke any change in the body weight gain of the dam or in its food intake throughout pregnancy, although they generate offspring with low birth weights and low levels of plasma glucose[217]. Thus, other endogenous factors, like excess maternal GC, might also be responsible for the low birth weight measured in PS neonates[15,314]. Indeed, since GC regulate the expression of GLUT transporters in the rat placenta[124], this elevated transfer of maternal corticosterone to the fetal compartment might modify the expression of GLUT1 and therefore the glucose transfer. In addition, a reduced expression and/or activity of placental 11β-hydroxysteroid dehydrogenase type 2 (11β-HSD2) could further favor the elevated GC

maternal transfer through the feto-placental unit, leading to IUGR (Fig. 2)[204]. This enzyme unidirectionally converts the endogenous cortisol (corticosterone in rodents) into its inactive metabolite cortisone (or 11-dehydroxycorticosterone in rodents)[293]. Thus, within the feto-placental unit, the 11β-HSD2 protects the fetus from an excessive transfer of maternal GC. Moreover, a reduced activity of placental 11β-HSD2 has been described in human IUGR pregnancies and low birth weights have been documented in patients bearing mutations of the *11β-HSD2* gene[210,219]. Similarly, inhibition of placental 11β-HSD2 in gestating rats by the specific inhibitor carbenoxolone reduces birth weights and leads to impaired glucose tolerance in the adult offspring[190]. In addition, the mating of heterozygous mice for a null allele of the *11β-HSD2* gene (11β-HSD2(+/–)) results in homozygous 11β-HSD2(–/–) offspring that have lower birth weight than their 11β-HSD2(+/+) littermates[140]. Lastly, a prenatal treatment with synthetic GC-like dexamethasone (DEX) has been shown to retard fetal growth and reduce birth weight in various mammalian species[294]. The understanding of GC impact in the programming of long-term diseases is of highest interest since synthetic GC are commonly used in human therapeutics in women at risk for preterm delivery. This treatment aims to promote fetal lung maturation, allowing the baby to breathe after delivery[106]. However, this medical treatment might not be devoid of potentially long-term deleterious side effects for the unborn child. Altogether, these data further highlight the deleterious consequence of an increased transfer of PS-induced excess maternal GC through the feto-placental unit.

## Neuroendocrine Control of Growth Axes

First, besides the feto-placental transfer of nutrients, GC also regulate the expression of metabolic hepatic enzymes, such as phosphoenolpyruvate carboxykinase (PEPCK), which catalyses a rate-limiting step in gluconeogenesis. In fact, elevated expression of PEPCK and increased activity of this enzyme are observed in newborn rats that had been prenatally exposed to high levels of GC[229]. Overexpression of PEPCK in rat hepatoma cells alters insulin inhibition of gluconeogenesis and transgenic overexpression of PEPCK in the liver impairs glucose tolerance[278,333]. Thus, elevated maternal GC might directly result in a dysfunction in the hepatic glucose pathway, leading to long-term hyperglycemia and insulin resistance. In this view, both restraint maternal stress and injection of synthetic GC during late gestation have been shown to induce hyperglycemia and glucose intolerance in adult or aged animals[185,229,334]. In addition to its function of nutrient, glucose also promotes maturation of glucose-stimulated insulin secretion in cultures of rat pancreatic islets[81], thus suggesting that malnutrition may retard the functional maturation of islets in PS offspring. In both humans and rats, the growth of pancreatic islets mainly takes place during the perinatal period. β-pancreatic cells express GR as early as from gestational day 12 in rats[165]. Thus, impaired pancreatic β-cell development may cause a lasting reduction in the insulin-secretory response that could later lead to an altered insulin secretory response to glucose at adulthood[7]. Indeed, PS reduces pancreas weight and impairs pancreatic β-cell development by reducing β-cell mass[25,107,108,204]. These disparate effects of gestational stress on glucose metabolism and insulin action, particularly noticeable in the case of food restriction, alter the regulation of hepatic glucose production and the suppression of adipose-tissue lipolysis, consistent with a state of insulin resistance in the adult or aged offspring. The better glucose tolerance observed in young PS animals as compared to aged rats seems to be due to an increased sensitivity to insulin; indeed glucose plasma concentrations are reduced in young PS rats as compared to controls[298]. However, an age- and gender-dependent loss of glucose tolerance in PS individuals is noticed in various studies, leading to frank diabetes more rapidly than in controls. Nonetheless, the mechanisms underlying these metabolic changes throughout life still remain unknown[237].

Second, insulin also possesses other growth-supporting activities on fetal tissues. Fetal growth is therefore strongly dependent on the interaction of the following hormones: insulin, growth hormone (GH), and insulin growth factor (IGF)[141]. In fact, *in vivo* fetal concentrations of IGF-1 and IGF-2 are positively correlated to birth weight in different species[6,90,175]. Animals in which genes encoding IGF-1, IGF-2, and IGF receptors have been deleted present a reduced body weight at birth compared to wild-type animals[11]. Similarly, IUGR has been reported in a patient with a partial homozygous deletion of the IGF-1 gene[373]. Interestingly, IGF-1 levels are highly regulated by nutritional factors in fetuses[90,232]. Thus, IGF-1 produced by the fetal liver and other tissues like the placenta appears to be the dominant fetal growth regulator in late gestation[179,180]. In the fetus, insulin mediates the hepatic IGF-1 production. In this view, insulin deficiency reduces levels of plasma IGF-1, but not of IGF-2, in the sheep fetus[115]. Thus, fetal insulin and IGF-1 levels are positively correlated and act synergistically to enhance accumulation of glucose and amino acids in fetal tissues[89,236]. Indeed, a deficit in the transfer of glucose or amino acids from the maternal to the fetal compartment in a context of chronic stress may alter the function of the insulin-GH-IGF axis. However, in late gestation, despite the paucity of liver GH receptors, GH-inducible genes are expressed within the fetal liver, suggesting that GH may be physiologically active before term[253]. In fact, abnormalities in the GH-IGF axis are commonly described in growth-retarded fetuses and neonates[139,346], although various effects have been described depending on the intensity of the gestational stress. Hence, a decrease in IGF-1 levels has been documented in cases of mild-to-severe gestational stress, whereas no effects on IGF-1 but reduced IGF-2 levels have been reported in cases of more severe nutrient deprivation[90]. In addition, IGF-1 levels are more readily affected by the fetal hormonal environment, such as insulin, thyroxin, and GC, than are IGF-2 levels. The surge of fetal corticosterone is essential to initiate the switch from GH-independent local production of IGF *in utero* to GH-dependent hepatic production of endocrine IGF-1[90]. Thus, fetal overexposure to GC may prematurely activate the fetal growth axis, resulting in a GH-dependent production of IGF, subsequently inducing an altered somatic development. Several studies reported abnormalities in GH secretory profiles and mean plasma IGF-1 levels, with a resistance to GH and IGF-1 effects[116], although discrepancies might occur with the PS procedure used. For instance, in a rat model of maternal protein restriction throughout pregnancy, no variation in GH secretory profiles were found between PS and control rats at any age, despite a consistent reduction in birth weight and a failure of catch-up growth[225]. Conversely, in the model of maternal restraint stress, we observed a decrease of fetal plasma GH without any change in IGF-1 levels[204]. In this view, one can speculate that PS reduces fetal pituitary GH secretion and/or its hypothalamic control through effects of GC or other hormones in relation with maternal nutrition[257]. In humans, most observations in the field emerged from studies of children with IUGR who present persistent abnormalities of the GH-IGF axis, like lower serum concentrations of IGF-1, IGF-2, and IGF binding protein-3[66]. Moreover, IUGR is commonly associated with postnatal GH resistance[3]. Altogether, these data further demonstrate that animal models recapitulate several alterations of the GH-IGF axis documented in humans.

Third, the parallel organization of hypothalamo-pituitary-thyroid (HPT) and HPA axes converges in mobilizing energy stores for the harmonious development of individuals (Table 2). In particular, a decrease in thyroid hormones elicits a reduced energy turnover in the whole organism, leading to alterations of the reproductive function, thermogenesis, immune function, or growth processes. The ecological importance of thyroid plasticity has been underlined by Flier and collaborators[86] from rodent models: "*starvation rapidly suppresses T4 and T3 (thyroid) levels. The benefit of this suppression is clear: starvation represents a severe threat to survival, and, in rodents, the capacity to survive without nutrition is measured in days. Because thyroid hormones set the basal metabolic rate, a drop in thyroid hormone levels should reduce the obligatory use of energy stores.*" Similarly, several studies have reported evidences of a prenatal programming of the HPT axis. In fact, data obtained from both humans and animals indicate that decreased or increased levels of maternal thyroid hormones generate low birth weight progenies[252,331]. Moreover, impairment of the offspring thyroid system is generally described[47,258]. For example, alcohol consumption during gestation (Table 1) induced hypothyroidism in the rodent adult offspring evidenced by decreased levels of $T_3$ and elevated thyreo-stimulating hormone (TSH) compared to controls[370]. Furthermore, maternal GC were shown to impact the function of the offspring HTP axis since offspring of adrenalectomized gestating dams showed reduced birth weight, reduced adult hypothalamic thyroid releasing hormone mRNA levels, and increased plasma TSH[306].

These data point out additional evidences of a strong connection between HPA and HPT axes. Indeed, activation of the HPA axis leads to concomitant decreased production of TSH and inhibition of the peripheral conversion of $T_4$ to biologically active $T_3$[248,329]. Furthermore, variations in levels of thyroid hormones have deleterious effects on other endocrine systems. In sheep and pigs, maternal hypothyroidism decreases IGF-1 levels and is accompanied by fetal growth retardation paralleled with tissue-specific changes in *Igf1*, but not *Igf2* gene expression[87,88,176]. Hence, changes in *Igf1* gene expression mediated by maternal thyroid hormones might have a key role in regulating fetal growth, particularly in tissues such as skeletal muscles that normally account for 25–33% of fetal body weight at term[236]. However, consequences of thyroid hormones on placental development and *Igf* gene expression still remain poorly understood and deserve further investigations. Thus, a deficient HPT axis due to excess maternal GC might have a strong impact on fetal growth by acting on various targets. These endocrine dysfunctions might also augment the susceptibility of PS offspring to develop various metabolic diseases during adulthood[47,222].

**TABLE 2**
**Common Features between HPA and HPT axis**

| | HPA Axis | HPT Axis |
|---|---|---|
| Hypothalamic hormones | CRH | TRH |
| Pituitary hormones | ACTH | TSH |
| Targets | Adrenals | Thyroid |
| Final product | Cortisol (corticosterone in rodents) | 3,5,3'-triiodothyronine ($T_3$); thyroxine ($T_4$) |
| Binding protein | Yes (CBP) | Yes (TBG) |
| Cellular location of receptors | Nuclear (main) and membrane bound | Nuclear |
| Central negative feedback | Hypothalamus; pituitary; hippocampus | Hypothalamus; pituitary |
| Main function | Mobilization of energy | Mobilization of energy |

ACTH: adrenocorticotropin hormone; CBP: corticosterone binding protein; CRH: corticotropin releasing hormone; TBG: thyroid binding protein; TRH: thyroid releasing hormone; TSH: thyroid stimulating hormone; HPA: hypothalamo-pituitary-adrenal axis; HPT: hypothalamo-pituitary-thyroid axis

Altogether, long-term consequences of such fetal changes in the GH-IGF axis are not fully understood yet in terms of functional adaptation or diseases. However, PS-evoked alterations might appear as potentially beneficial for the short-term survival in an environment of shortage of nutritional resources. Conversely, when such changes emerge in a context of food abundance as in our occidental societies, they might ultimately lead to the development of diseases.

## Body Weight, Leptin, and Altered Development

Body weight and energy mobilization are also under the control of the adipocyte-derived hormone leptin[378]. The current understanding of leptin's biological functions varies from the regulation of excess body weight to broad effects on reproduction, hematopoiesis, angiogenesis, blood pressure, bone mass, lymphoid organ homeostasis, and T-lymphocyte systems. Indeed, leptin orchestrates complex biological pathways through its receptors, expressed both centrally and peripherally. Leptin deficiency or resistance can result in obesity, diabetes, and infertility in humans. Fasting as well as chronic undernutrition decrease circulating levels of leptin. Indeed, a lack of leptin triggers the sensation of hunger, thus prompting the individual to seek food and to eat, thus ensuring his/her immediate survival. In parallel, such a decrease also inhibits reproductive functions until the organism's fat stores are back to normal and able to supply enough energy for a potential gestation. Leptin is known to act on the brain, where it specifically activates hypothalamic anorexigenic neurons and inhibits orexigenic neurons, thus leading to a reduction of the food intake[291].

The prenatal programming of appetite-mediated ingestion can be viewed as the facilitation of food intake and survival in the newborn. In fact, gustative sensations (sweetness, bitterness, acidity) that mediate nutrient ingestion are nearly functional at term in various species[279]. In the rodent restraint gestational stress model, there are no changes in plasma levels of fetal leptin at term[185]. However, the administration of DEX during the last third of gestation leads to maternal hyperleptinemia paralleled with fetal hypoleptinemia; at adulthood, PS male offspring exhibit hyperleptinemia compared to age-matched controls associated with hyperinsulinemia[316]. Therefore, this hyperleptinemia may be a component of the cluster of metabolic abnormalities seen in the insulin-resistance syndrome. Although leptin inhibits food intake in adults, it is ineffective in the neonate rodent[263]. The inability of leptin to regulate food intake in rodent pups might be related to the immaturity of hypothalamic orexigenic/anorexigenic pathways. Hence, cerebral structures involved in the control of food intake achieve their development after birth[28,29]. Since GC and feeding behavior are strongly associated, alterations in the feeding behavior of PS animals during stressful situations might be related to dysfunctions of their HPA axis[41,111]. Indeed, early stress paradigms have been shown to reduce the daily food intake of young PS adult rats[245,334]. Conversely, a fasting period significantly increases hyperphagia in aged PS rats[185]. Aged PS rats exhibit reduced levels of leptin, but no change in the weight of several adipose tissues, which could be related to altered adipocyte metabolism[185]. In support of this hypothesis, prenatal administration of DEX results in increased GR expression and attenuated fatty acid uptake in adult visceral adipose tissue[50]. Moreover, maternal protein restriction augments the preference for high-fat foods in the adult offspring, leading to a greater degree of obesity than control animals[174]. In humans, as shown in the Dutch famine birth cohort study (2,414 term singletons born alive between November 1943 and February 1946 in Amsterdam), famine during pregnancy, whatever the gestational stage, results in glucose intolerance, higher atherogenic lipid profile responsible for coronary heart disease, and obesity, especially in women[277]. Likewise, the relative contribution of genetic (fetus) vs. environmental (maternal/placental) factors on growth was studied on monochorionic twins with intertwin birth weight differences and treated by laser coagulation for severe twin-to-twin transfusion syndrome[117]; leptin was shown to be not only an index of fetal fat mass, but also a determinant of fetal brain development. Finally, the lack of choline triggered by a food-restriction procedure might alter DNA methylation, gene expression, and associated changes in stem cell proliferation and differentiation, as recently reviewed by Zeisel[377]. Thus, besides the direct consequences of the gestational environmental context (maternal/placenta), one can hypothesize that PS might have more deleterious outcomes in modifying the structure of fetal genes, eventually leading to permanently altered physiological functions.

In conclusion, chronic gestational stress mainly alters intrauterine growth, thus leading to a low birth weight. Several neuroendocrine systems are involved in growth regulation whose dysfunctions might partly explain the emergence of metabolic disorders throughout life. Despite the general view of maternal GC as the main actors in the development of deficiencies in the offspring, the impact of other potential indirect factors still remains to be addressed. Furthermore, PS long-term consequences on the offspring might also depend on their feeding behavior after birth. A rapid catch-up growth may indeed lead to obesity, whereas a delayed catch-up growth could favor cardiovascular diseases. Despite the paucity of data in human beings, one can expect similar processes to occur. In terms of individuals' survival, we can wonder what would be the best response to adopt in order to be adapted to the environment without long-term deleterious effects. Perhaps, as suggested by Charles Darwin: "*it is not the strongest of the species that survives, nor the most intelligent, but the one most responsive to change.*"

## PRENATAL STRESS ALTERS SEXUAL DIFFERENTIATION

Among the physiological and behavioral systems sensitive to stress during gestation, the reproductive system or hypothalamo-pituitary-gonadotropic (HPG) axis may be easily affected. Indeed, when gestating dams undergo adverse circumstances, alterations in the reproductive capacities might occur in the offspring. The PS impact on the sexual organization seems to be determinant, especially if the gestational stress takes place between gestational days 15 and 20 in rats or between weeks 3 to 12 after fecundation in human beings. Numerous data gathered from studies conducted in different species, including humans, describe the impact of PS on sexual behavior, sexual hormone levels, and on the development of sexual brain areas.

### Sexual Behavior

The initial work of Ward[353] on rats clearly showed that PS is a potent disruptor of the normal course of sexual differentiation. In particular, it demasculinizes and feminizes the behavior of the male offspring. Indeed, when dams are restrained under bright light from days 14 to 21 of gestation, male PS rats display at birth a reduced ano-genital length and a lower testis weight compared to controls[204,299], which could predict an impaired sexual activity at adulthood[159]. In addition, the completion rate of testicular descent is 14% in 21-day-old PS rats and 64% in age-matched controls, thus demonstrating a reduced testicular descent in the PS offspring at weaning[300]. Moreover, at adulthood, PS males exhibit reduced copulatory capacities, impaired ejaculation, and high-quality female lordotic responses[353], which further underlines these PS-evoked alterations of sexual behavior throughout life.

Restraint gestational stress also disrupts sexually dimorphic behaviors displayed before puberty in the male progeny, like juvenile play[356], whereas no behavioral abnormalities are detectable in the female offspring[354]. However, using a more intense paradigm combining heat, restraint, and bright light, Herrenkohl[135] showed that PS alters fertility and fecundity in the female offspring. Besides, PS females present alterations of their estrus cycles as well as of ovarian and uterine weights at autopsy although their sexual receptivity appears intact[136]. Hence, these PS animals give rise to less successful conceptions, more spontaneous abortions as well as vaginal hemorrhaging, longer pregnancies, and fewer viable infants than females born from nonstressed dams[135]. Moreover, Frye and Orecki[100] recently reported that PS females display behavioral inhibition in mating situations and decreased intensity of the typical female copulatory posture (so-called lordosis). In this view, PS-induced behavioral perturbations appear to be related to the intensity and the nature of the gestational stressor, although female progenies seem to be more resistant to the sole effects of gestational stress as compared to males[100].

Besides the consequences of gestational stress on the sexual behavior of first-generation descendants, it is important to consider the transmission of these PS-programmed alterations through generations. Indeed, the impact of PS on reproductive behaviors might persist through two or three generations in rats and in hamsters, respectively[144,255]. In rats, individuals from the second generation grow more slowly, thus giving rise to PS adult animals of both sexes being permanently smaller than their control counterparts. However, at the second generation, HPG alterations are only present in male rats of the PS descent; in particular, these animals display higher concentrations of plasma progesterone as compared to control males, whereas PS females give rise to litters of the third generation that do not differ from controls[255]. In hamsters, food restriction during gestation has long-term consequences on sex ratios at the third generation with similar postpartum mortality rates for both male and female pups[144].

Finally, gestational stress has also been suggested to influence sexual differentiation in humans. In fact, Dorner and coworkers, studying men from the German Democratic Republic born during the Second World War and the early postwar period, i.e., between 1941 and 1947, showed that such a gestational stress may represent a possible etiogenetic factor of homosexuality later in life in the male progeny[79,80]. However, one has to keep in mind the common pitfalls of studies conducted on human populations, often due to the lack of well-documented information on pregnancies and the small size of the cohorts studied. In this view, recent data gathered from a cohort of 13,998 pregnant women who gave birth to a total of 14,138 children highlighted the absence of PS influence on the development of gender role behavior in boys and the relatively little influence on this parameter in girls[138]. Further studies should be conducted in order to compensate the lack of consistent data in humans and specify the actual outcomes of PS on sexual behavior.

Altogether, the extent of PS-induced alterations of the HPG function greatly depends on the intensity of the gestational stressor to which the gestating dam is exposed. These impairments may impact the reproductive success (the so-called fitness) of the species. Indeed, an atypical behavior before and/or during mating can dramatically reduce the success of breeding. Such behavioral modifications could favor the fitness when the environment in which organisms are living is noxious, harmful, or dangerous for the survival of the second-generation progeny. As mentioned earlier, PS consequences might be either an advantage or a drawback according to the life milieu in which the offspring has to grow.

## Sexual Hormones

It is widely known that hormones drive many behavioral schemes and sexual behavior is not an exception. In this view, one can assume that PS-induced modifications of sexual behavior in species might be related to alterations of the HPG axis and its hormonal secretions. In fact, in a physiological context, sexual organization occurs at a specific sensitive period of development during which the presence or absence of sexual hormones may have determining effects on the biological sex of the individual. In male rat fetuses, a massive peak of plasma testosterone arises between the $18^{th}$ and $19^{th}$ intrauterine day and a second one during the first few hours after birth. Testosterone is synthesized in the testis as early as day 17 of gestation. This synthesis is then followed by a large peak between the $19^{th}$ intrauterine and $4^{th}$ postnatal days and by a smaller one around postnatal days 14–15[60,65,367]. In female fetuses, however, testosterone is produced by gonads as well as placenta and/or fetal adrenals, and its plasma concentrations remain constantly elevated throughout gestation[143]. The male testosterone peak occurs concomitantly with the critical timing of brain sexual differentiation. On the contrary, in females, no detectable amount of steroids has been described in the ovary during the perinatal period, suggesting that female gonads might not be involved in the early period of brain sexual differentiation[65]. In nonhuman primates as well as in human beings, prenatal testosterone seems to exert a similar influence on sexual differentiation. Indeed, secretion of testosterone from fetal testis Leydig cells during the first half of primate gestation modifies the organization of both the reproductive system and cerebral structures selecting and controlling a proper sexual behavior[205]. Lastly, one cannot undermine the influence of the intrauterine position for litter-bearing species since a female fetus positioned between two male fetuses is exposed to higher levels of androgens than a female surrounded by two other female fetuses[105,348]. In particular, female fetuses developing between two males tend to be masculinized in their anatomy, physiology, and behavior at adulthood, whereas female fetuses developing without adjacent males tend to show more feminized traits as adults. Thus, permanent alterations of hormone levels, reproductive organs, secondary sex ratios, and susceptibility to endocrine disruption are observed in the females surrounded by males during gestation. Some of these effects are similar to the influence of PS on adult phenotypes[282].

In the context of gestational stress, physical as well as emotional PS have been recognized to disrupt the reproductive function of the male progeny in suppressing the fetal testosterone peak on gestational days 18 and 19[305,355,358]. Similarly, a treatment of gestating dams with DEX[170] or hydrocortisone[246] gives rise to PS male rats with reduced testosterone levels at adulthood. In addition, it has to be noted that PS reduces concentrations of not only rat fetal testicular testosterone, but also fetal pituitary luteinizing hormone (LH)[300]. However, depending on the gestational stress paradigm, discrepant results might be obtained. In this view, male progenies generated from dams that were given a liquid alcohol diet in late gestation exhibited no modifications of plasma testosterone and LH levels[357]. Finally, adult circulating LH levels (known to be an index of sexual arousal) are lower in PS males exposed to a sexually receptive female[161], thus reinforcing the deleterious impact of PS both on sexual behavior and hormone levels. However, a testosterone propionate treatment given at birth to PS males counteracts some effects of the restraint maternal stress on the endocrine system and sexual behavior of the offspring[247]. Moreover, this treatment prevents the reduction of the ano-genital distance observed in pups aged 22 days as well as the decrease in testosterone levels at adulthood, thus leading to an improvement of sexual performances and reversing PS deleterious effects.

Thus, whatever the gestational stress paradigm used, male sexual behavior in adult mammals requires an adequate physiological functioning of the HPG axis *via* the secretion of proper testosterone levels at specific critical developmental set points. In this view, PS-induced altered testosterone levels observed both in testis and plasma of the male progeny are responsible for the altered sexual behavior later expressed; however, other possible factors have to be taken into account, such as alterations in brain responses to androgenic activation of male sexual behavior. Among other factors to be considered, excess maternal GC might also have potential deleterious effects. Indeed, corticosterone exerts adverse effects on the steroidogenesis in Leydig cells that can be attenuated by the presence of both types 1 and 2 11β-HSD[109].

## Sexual Brain Differentiation

Besides their involvement in tuning sexual behavior as underlined in the preceding paragraph, another fundamental role of sexual hormones during fetal and perinatal development is to drive the differentiation of specific brain areas controlling the HPG function and thus sexual behavior. Indeed, during these critical developmental periods, the brain is highly responsive to sexual hormones secreted by the fetus. These lipophilic steroid molecules readily cross the blood brain barrier and act on specific receptors located in various parts of the brain. Indeed, such receptors are found in the hypothalamus, more specifically in the preoptic area, the ventromedial, and the arcuate nucleus, as well as in limbic structures, such as the hippocampus, amygdala, septum, accumbens nucleus, and bed nucleus of the stria terminalis[303]. The effect of exogenous or endogenous testosterone on its receptors is mediated by its conversion to estrogen by the enzyme aromatase expressed within specific neurons[208]. Therefore, when aromatase or sufficient concentrations of testosterone are available during development, the brain can adopt a masculinized (and thus defeminized) organization, leading to a male wiring of the brain. Blocking the androgen pathway with antiandrogens does not affect the organization of male-typical mounting behavior[121,122], suggesting that the male rat brain is primarily masculinized by the estradiol generated from the aromatization of testosterone. In addition, some nervous structures are sexually dimorphic, like the spinal nucleus bulbocavernosus that contains motoneurones controlling copulation, or the sexually dimorphic nucleus of the medial preoptic area (SDN-POA) located in the hypothalamus and involved in sexual behavior and HPG regulation[113]. Both nuclei contain a greater number of cells in males than in females[5,118,119]. Their size strongly depends on gonadal steroids secreted during fetal development[118,148]. Most studies related to the cellular effects of gonadal steroids on sexually dimorphic cerebral structures reported that testosterone decreases the number of pyknotic cells in the developing spinal nucleus bulbocavernosus and reduces the incidence of apoptosis in the SDN-POA in the early postnatal period[71,227]. Additionally, the absence of testosterone or its metabolites feminized sexually dimorphic structures[272]. In fact, PS has been found to alter the sexual dimorphism of brain structures in male rats; these animals exhibit a diminished number of neurons in the spinal nucleus bulbocavernosus[123] and a decrease in c-fos activity within the medial preoptic area[146]. However, these PS-induced effects on neural differentiation are specific since several structures like the medial amygdala are not affected by PS-evoked alterations of the prenatal testosterone peak[157]. Additionally, a transient elevation in mRNA expression of the hypothalamic estrogen receptor is observed in PS male rats, further underlying the role of PS in the deviation of sexual development towards a feminized profile[133]. Moreover, the neonatal hypothalamic aromatase is decreased in PS males only, whereas no change has been noted in the amygala in either sex[221].

Taken together these observations support a pivotal role for androgens in the demasculinization process documented in the PS male progeny. Indeed, the PS-induced reduction in the production or aromatization of testosterone in males during the perinatal period of sexual differentiation may lead to a transient up-regulation of unstimulated estrogen receptors. In conclusion, alterations of the sex steroids system might influence sexual behaviors as well as certain physiological functions of individuals throughout their life. Moreover, exposure of the developing brain to severe and/or prolonged stress may result in hyperactivity/hyperreactivity of the stress system that is often paralleled with a temporary or prolonged abrogation of reproductive functions. Thus, PS occurring during the period of sexual differentiation may result in impairments of various physiological processes with behavioral and/or somatic sequels.

## PRENATAL STRESS AMENDS STRESS RESPONSE

Adaptation to daily changing situations requires the ability for organisms to cope adequately with the "*physical or perceived threats to homeostasis*" (according to the operational definition of stress from Pacak and Palkovits[239]). For this purpose, a complex network of several interrelated central and peripheral circuits sustains a permanent cross-talk between neurological and endocrine messages. Thus, the stress response is characterized by an enhanced adaptation that mobilizes energy stores to re-establish the homeostatic balance. Stress responses always recruit the activation of the autonomic nervous system, especially its sympathetic branch and the HPA axis, giving rise to the release of catecholamines and GC, respectively (Fig. 1A). The general purpose of these activations is to enable the individual to mobilize its resources towards action; in other words, such autonomic driven modifications are initiated to favor the so-called "*fight-or-flight reaction*".

### General Physiology of the Stress Response

In physiological conditions, any change in the environment first elicits a cognitive evaluation through different cerebral circuits directly or indirectly connected to the limbic system. If the stressor is perceived as a threat, it then triggers the response to stress. At the central level, this response recruits sympathetic preganglionic neurons located in the spinal T2-L1 segments[265] that are under the direct or indirect control of various brainstem areas (ventrolateral medulla, locus coeruleus, periaqueductal gray) and/or superior cerebral structures (hypothalamus, prefrontal cortex, amygdala)[194,284,324]. The activation of the HPA axis principally involves the parvocellular part of the hypothalamic PVN whose neurons release CRH and AVP in the hypophyseal portal blood (Fig. 1A). Since AVP potentiates CRH actions, it is considered as an important mediator of the HPA response to chronic stress. In turn, both neurohormones stimulate the secretion of ACTH from the anterior pituitary lobe via CRH type 1 and AVP type 1b (V1b) receptors[162,266,271]. Plasma ACTH then triggers cells of the adrenal cortex to release steroid hormones, namely GC and mineralocorticoids[287], which results in the enhancement of metabolic processes (glycogenolysis, gluconeogenesis, lipolysis, proteolysis, etc.), the modification of immune responses, and the alteration of the fluid and electrolytic balance. Beyond their peripheral actions, GC play a key role in the primary negative hormonal feedback loop within the HPA axis *via* two types of receptors, MR and GR[288]. The PVN is also controlled by neural structures of the brainstem and the limbic system (Fig. 1B). Thus, this hypothalamic nucleus is at the interface between the autonomic regulation and the integration of emotional features related to the stressful events.

These tight connections between the HPA axis and the sympatho-adrenal system have been selected through complex evolutive processes in order to produce and control adequate physiological and behavioral responses to stress. In this view, alterations of any component of one or both circuits might result in dysfunctions, especially if such deleterious events occur during critical periods of development, which has been documented in numerous studies[235,376]. One should, however, keep in mind that in cases of extreme environmental variations causing excessive maternal stress, the resulting activation of both the HPA axis and the sympatho-adrenal system provokes uterine relaxation, thus leading to miscarriages. When facing such extreme environmental threats, the survival of the sole maternal organism is favored, for a later increase of the species' fitness.

## Prenatal Stress and the Programming of the HPA Axis

Numerous studies in various species report modifications of the HPA axis reactivity of PS offspring and thus provide evidences of a prenatal programming of the response to stress and associated disorders. However, depending on the timing at which gestational stress occurs, PS-evoked maternal hormones, such as GC, might diffuse through the feto-placental barrier and thus impair the fetal development (Fig. 2). In response to maternal changes, the developing individual adapts, in turn, its own physiology to increase its own survival in a disturbed gestational environment. These fetal physiological adaptations initially aimed at promoting the immediate survival of the individual could, in the long term, increase stress responses both at neuroendocrine and behavioral levels.

### *Neurobiological Consequences of Prenatal Stress*

The maturation of the mammalian HPA axis is highly species-specific and usually occurs during mid-to-late gestation. In physiological conditions, i.e., when mothers are not stressed during gestation, an exponential increase in stress hormones in both the mother and the fetus arises at the end of gestation to boost maturation of fetal organs (heart, lung, brain, etc.) and the preparation for parturition[43]. Conversely, a chronic gestational stress consisting of repeated short-lasting sessions induces an anticipated maturation of the fetal HPA axis. In fact, restraint gestational stress in rats from embryonic day 15 (E15) to E17 (term ~ E21; Table 1) modifies the morphology of PVN fetal neurons by enhancing cell differentiation, increasing the branching and the total length of processes from the cell body, as well as by augmenting CRH mRNA expression[102]. However, extending restraint stress sessions to 240 min (instead of 30 min) has a neurotoxic action on the fetal PVN as indicated by an increased number of apoptotic neurons[102]. Since GR are expressed by E16 in the fetal rat PVN, such deleterious consequences on the development of the fetal PVN are thought to be primarily mediated by the PS-induced excess release of maternal GC[49]. Similarly, PS affects the development of the hippocampus in an intensity-dependent manner since a short-lasting mild PS enhances neonatal neurogenesis and processes differentiation of hippocampal neurons, whereas a long-lasting severe stress impairs their morphology[101]. Recently, Van den Hove and coworkers reported a drastic inhibition of cell proliferation (–58%) and an increased activity of caspase-3, a pivotal mediator of apoptotic pathways, in the hippocampus of 1-day-old PS pups, associated with a decrease in hippocampal brain-derived neurotrophic factor (BDNF) levels in 5-day-old PS rats, thus providing additional arguments in support of PS-induced neuroplasticity[340]. In addition, they reported a 25% reduction in the hippocampal astroglial-specific neurotrophic factor (S100B), known to play an important role in the physiological brain development[339]. Other evidences support a PS-induced dysfunction of the adult hippocampus. Indeed, adult PS offspring exhibit a decrease in the hippocampal weight[320], a decrease in the synaptic density[131], a reduced number of granule neurons associated with a marked reduction of hippocampal neurogenesis throughout life[182], and a lowered density of nitric oxide–producing neurons in the fascia dentata and Ammon's horns, possibly impairing hippocampal neurogenesis and long-term potentiation[332]. In addition, PS affects other limbic structures like the amygdala, known to play a key role in the control of emotional behaviors such as anxiety and fear. In particular, PS increases the number of neurons and glia in the lateral amygdaloid nucleus[283]. These detrimental cerebral changes may partly explain the later increased susceptibility of PS individuals to mood disorders (see "PS AND THE PROGRAMMING OF DISEASES OF THE ADULT" below). Finally, fetal brain injuries have also been described in humans in cases of impairments of oxygen delivery when pregnant women develop diseases like coagulation disorders, anemia, metabolic disorders, or cardiovascular collapse[260]. Altogether these data highlight the deleterious impact of chronic and intense maternal stress on the development of neonatal brain structures involved in the HPA axis maturation and function.

## *Neuroendocrine Consequences of Prenatal Stress*

The above-mentioned studies suggest that PS alters the organization of the fetal brain as well as plasma levels of various hormones. Indeed, fetal abnormalities in the brain development are thought to be prompted by impairments of the excess of maternal GC release occurring in stressing situations. In a nondisturbed gestation (i.e., physiological condition), low levels of endogenous maternal GC reach the fetus, this transit through the placenta being tightly modulated by 11β-HSD2 as already detailed (see "Role of Nutrients in Growth" above.)[33]. Recently, we showed a reduced expression of placental 11β-HSD2 and a subsequent decreased activity of this enzyme in the feto-placental unit of dams submitted to restraint PS that were paralleled with a decrease in the fetal adrenal weight[204]. These modifications in 11β-HSD2 expression and activity may contribute to an increased GC transfer through the feto-placental unit. This surge of maternal GC may thus impact the fetal development of brain GR and MR. In rats developing within physiological conditions, GR mRNA can be detected throughout the hippocampus, hypothalamus, and pituitary by E13 with increasing levels up to term (E21), whereas MR mRNA is detectable in the hippocampus by E16–17[31,49,311]. However, PS is reported to reduce the basal number of both hippocampal GR and MR in the adult progeny[202,270]. More recently, the effects of antenatal GC on the ontogeny of hippocampal corticosteroid receptors were investigated in human and mouse hippocampus. Indeed, a single antenatal administration of DEX at E15.5 transiently alters the levels of MR mRNA in the mouse hippocampus, whereas no changes are detected on the human hippocampus at the third trimester of pregnancy[226].

Furthermore, both basal levels of GC and their secretion under stressful conditions are modified in the PS progeny. Indeed, in a rat model of restraint PS (Table 1), the adult offspring display alterations of the circadian rhythm of corticosterone at rest and a prolonged elevation in plasma GC levels following an acute stress[164,203]. In addition, an accelerated aging of the HPA axis has been documented in PS animals; indeed, the delayed return to basal levels of GC after an acute stress seen in 4-month-old PS rats is observed in control animals only from the age of 24 months[336]. It might be of interest to note that such an age-related increase in circulating GC levels has also been observed in humans during aging[201]. Thus, PS reduces the GC negative feedback on the HPA axis, this effect being mediated by the reduced expression of hippocampal corticosteroid receptors[132,202,203]. Moreover, we recently showed that PS modifies the expression of Fos protein in brain areas involved in the negative feedback of the HPA axis, such as hippocampus and locus coeruleus[347]. Alterations of this negative feedback might be related to alterations in the GABAergic PVN regulation and/or a modified norepinephrine release from the brainstem. In fact, reduced norepinephrine levels and an increased norepinephrine turnover have been described in the hippocampus and within the locus coeruleus, respectively[220,321].

Besides GC, other factors can influence the fetal development, such as CRH, ACTH, or other pro-opiomelanocortin–derived peptides. Placental CRH is believed to coordinate and control the physiology of parturition *via* its actions on the fetal HPA axis as well as on other tissues[38]. In rats, the fetal HPA axis can respond to variations in maternal ACTH, CRH, and corticosterone around E17[15,27]. In humans, a negative correlation has been documented between levels of maternal circulating CRH and the length of gestation; preterm delivery is correlated with high maternal CRH levels, whereas no change is noted in fetal CRH[193,350]. However, dissociation between maternal and fetal plasma CRH levels occurs in the case of acute stress caused by intrauterine needling characterized by an increase in the fetal CRH levels[114]. The precise outcome of excessive CRH levels on the neuronal function and/or integrity of fetal brain still remains largely unknown, although several studies investigating behavioral consequences of PS on the activation of CRH receptors in the hippocampus and other limbic regions, like amygdala, have reported learning and memory impairments[10,365] Other HPA axis factors differentially evolve between the mother and fetus; maternal ACTH levels decrease with gestational age, while fetal ACTH levels increase[193] However, due to the paucity of available investigations, the importance of other HPA axis hormones in mediating PS adverse consequences on neuroendocrine functions still remains to be assessed.

## Behavioral Consequences of Prenatal Stress

Besides PS-induced endocrine and neurobiological modifications, an increased behavioral responsiveness to stress is also commonly described in the young and adult PS offspring. Indeed, PS animals exhibit a higher behavioral emotionality in several stressful situations as evidenced in the elevated plus maze[335], in a situation of acute restraint stress[202], when exposed to novelty[40,75], or in the forced swim test[215]. Moreover, when exposed to a brightly lit, large, and intimidating open field, PS rats display less exploratory activity than controls[256]. Likewise, PS rhesus monkeys exposed to a stressful environment display a reduced exploratory activity and more stereotypic behaviors[289]. Although studies conducted on human populations are subjected to certain already-mentioned common pitfalls, the literature on human beings nevertheless sustains the hypothesis of the programming of the fetal HPA axis by gestational stress, such as psychosocial stress (noise), familial and/or marital discord, death of a parent or spouse, or war[9,338,365]. Recently, O'Connor and coworkers[230] demonstrated for the first time a significant link between prenatal anxiety in late pregnancy and elevated morning cortisol in the preadolescent offspring in a study based on the Avon Longitudinal Study of Parents and Children. Moreover, this survey reveals a correlation between behavior and gender in 4-year-old children. Among disturbances observed, boys display hyperactivity and inattention, whereas girls exhibit emotional problems.

In conclusion, altogether these data underline the potential impact of PS on the organization of the HPA axis and on a number of brain areas involved in its regulation. These neurobiological and endocrine alterations might then generate maladapted behaviors in the long term that would lower the ability of individuals to face stressful situations. If excess GC arise before and/or after critical sensitive periods of development, this could generate only transient effects among which the beneficial outcome would prevail over potential harmful consequences. However, when such excess secretions parallel a critical time-point of fetal development, especially of cerebral structures, long-lasting consequences may coincidentally be programmed that might be deleterious for the individual-to-be and later lead to diseases of the adult.

## Prenatal Stress and Programming of the Sympatho-Adrenal System

Numerous physiological and emotional behaviors, including locomotion, exercise, flight, or aggression, as well as passive coping responses, require the concomitant activation of the HPA axis in parallel with motor and sympathetic efferent pathways. Tract-tracing studies underline the involvement of a subset of PVN sympatho-motor neurons that contain either AVP or oxytocin neural terminals[158]. Thus, besides their role in the regulation of the HPA axis, PVN neurons also mediate the regulation of the autonomic nervous system. In fact, through its reciprocal connections with brainstem nuclei (Fig. 1), the PVN is considered as a prime candidate within the forebrain for mediating sympathetic outflows during physiological states as well as heart failure[242]. This modulation recruits glutamate inputs that target PVN presympathetic neurons and are tonically inhibited by a GABA(A)-mediated mechanism[189]. Furthermore, the stimulation of PVN elevates serum norepinephrine *via* a neural mechanism[206]. Additionally, vasopressinergic PVN neurons also participate in the sympathetic regulation. Indeed, they are activated during increases in plasma osmolality and might elicit sympatho-excitation through their projections on spinal neurons expressing V(1a) receptors[4].

Like the HPA axis, the sympatho-adrenal system might also be affected by gestational stress, the reported effects of which vary according to the PS paradigm used[376]. For example, the adult offspring of dams submitted to unpredictable stress during gestation do not differ from controls in their resting main arterial blood pressure, heart rate, or epinephrine and norepinephrine plasma levels, whereas they exhibit an enhanced activation of the sympathetic nervous system in response to an acute footshock stress, as reflected by increased norepinephrine plasma levels[364]. Similarly, a prenatal exposure to the opiate morphine generates a sympatho-adrenal hyporesponsiveness to an ether inhalation stress in adult offspring[169], whereas under resting conditions, both adrenal norepinephrine and epinephrine are reduced in these PS rats although plasma epinephrine levels are increased compared to control rats[82]. These data clearly indicate that (1) PS can alter the development of the sympatho-adrenal system and (2) the autonomic response in the adult offspring depends on the nature and/or intensity of the stressor applied during gestation and/or adulthood. Moreover, these PS-evoked sympathetic modifications do not equally affect the whole sympatho-adrenal system, but appear to be rather restricted to cardiovascular components. For example, restraint PS has opposite effects on hyperthermic responses of adult offspring submitted either to an acute restraint stress or to an injection of lipopolysaccharide[129]. Indeed, these PS rats show no variation in plasma catecholamine levels either at rest or in the two stressful situations compared to controls. The mechanisms responsible for such opposite effects are not yet fully understood. However, one can hypothesize that a dysfunction in the adrenal organization, for example, in enzymatic activities, could alter catecholamine secretions. In this view, in a rodent model of innate anxiety, we recently showed a decrease in enzymes involved in the synthesis of catecholamines in rats with the highest anxiety behaviors[285]. However, besides a potential disorganization of the adrenals, alterations in the central control of the sympatho-adrenal activation might be responsible for the above-reported dysfunctions. The function of adrenals is controlled by thoracic sympathetic preganglionic neurons whose activity is influenced by various brain areas. In fact, PS-induced alterations in the PVN regulation by GABA, serotonine, or norepinephrine inputs might affect plasma catecholamine levels as well (Fig. 1B). In addition, as already mentioned, PS modifies the morphological organization of the amygdala[283], a limbic structure essential for regulating emotional and autonomic responses, and increases its CRH content which might induce a greater sympatho-adrenal activation to emotional stress in the PS offspring[63].

Finally, through these effects on the release of adrenal catecholamines, PS modifies several cardiovascular parameters. Indeed, PS offspring generated from prenatal hypoxic stress in rats and later exposed to stressful conditions at adulthood exhibit increased blood pressure and a higher variability of both blood pressure and heart rate as compared to controls[249]. Similar data were obtained with a paradigm of restraint gestational stress in rats[147] in which long-term effects of an acute stress were evidenced on cardiovascular responses of PS animals with a greater effect in males than in females. Indeed, PS females showed a greater increase in systolic arterial pressure, an increased variability of blood pressure, and a delayed heart rate recovery following return to the home cage than did PS males[147]. Likewise, gestational undernutrition induces an elevation of the diastolic blood pressure and the heart rate in PS offspring during the waking phase of their cycle[327]. Thus, maternal undernutrition may program cardiovascular dysfunctions throughout life span[173]. In particular, a PS-induced high blood pressure might ultimately lead to arterial hypertension. Taken together, these data gathered from experimental models reinforce Barker’s initial hypothesis of a prenatal programming of coronary heart diseases in human beings[17]. However, further investigations are needed for identifying the putative mechanisms underlying such PS-evoked alterations, like potential structural changes in the blood vessels and/or changes in the central or local control of the vasculature. Moreover, the paucity of the available data on the programming of the sympathetic and parasympathetic nervous system in humans underlines the need of conducting such studies despite their difficulties[250]. Nevertheless, most investigations in the field in human beings focused on PS consequences on HPA axis and birth weight, often documenting sex-dependent alterations in blood pressure and heart rate when individuals are submitted to psychosocial stress[153,250]. Thus, a disturbed gestational environment can exert long-term effects at various levels on the autonomic nervous system in human beings that might program several cellular changes especially within the cardiac tissue (for example, variations in the cardiomyocytes number) and in coronary vessels[197].

In conclusion, dysfunctions of numerous neuroendocrine systems caused by various gestational stresses might lead to physiological changes in the adult offspring. Since PS indirectly alters cardiovascular risk factors, such changes may predispose the PS progeny to cardiovascular diseases. Thus, the combination of enhanced stress susceptibility in adult PS individuals and the daily psychosocial stressors to which people are exposed may constitute a significant component of the disease risk in human populations.

## PRENATAL STRESS AND THE IMMUNE FUNCTION

In response to the permanent threat of being killed by pathogens, primitive eukaryotes have consistently evolved antipathogen devices aimed at favoring their immediate survival in face of a hostile environment, therefore ensuring the transmission of their genes to the following generations. Inherited from these ancient times when life mostly meant primary survival and constant fight between unicellular organisms, the first line of host responses to pathogen invasion in pluricellular organisms is the innate immune defense, a nonspecific mechanism involving macrophages, dendritic cells, and natural killer (NK) cells, as well as soluble factors such as cytokines or proteins of the complement system[2,112]. In addition to these ancestral mechanisms, jawed vertebrates have developed a more complex adaptive immunity that is highly specific, selective, remembered, and tightly regulated, unlike innate immune components. This acquired immunity implicates immune cells expressing antigen-specific receptors and is thus primarily mediated by B and T lymphocytes as well as humoral components of the immune system[59,241]. In this evolutionary perspective, the immune system appears as one of the oldest strategies developed by living metazoans to adapt the physiology of organisms to potential environmental threats.

### Cross-Talk between Nervous, Immune, and Endocrine Systems

In vertebrates, and more especially in mammals, a permanent communication between nervous, immune, and endocrine systems underlies homeostasis within organisms (Fig. 3A). In particular, the HPA axis and the autonomous nervous system tightly interact with the immune system to generate adaptive responses to stressful situations. Indeed, on one hand, immune cells express receptors for both hormones and neurotransmitters[280]; triggering of these receptors will therefore result in the modulation of the immune reactivity. On the other hand, nerve cells can respond to the molecular mediators secreted by immune cells, namely cytokines, that prompt many physiological reactions to infection like fever *via* the expression of their specific receptors[58,67]. Furthermore, an inadequate communication between neuroendocrine and immune systems has been suggested to contribute to the physiopathology of disorders associated with immune alterations, such as the chronic fatigue syndrome or autoimmune diseases (Fig. 3B)[149,155]. Finally, an increase in GC levels has been reported during the course of an immune response[23,134,270], further supporting the concept of reciprocal influences of the central nervous system, the immune system, and the endocrine system on each other, mediated by both neurohormones and cytokines.

### Gestational Stress and Development of the Immune System

The *in utero* environment is critical for initiating the ontogeny of several physiological systems, including the immune surveillance. In physiological conditions, the immune system is progressively built during embryonic and fetal development. In particular, humoral components of the immune system develop earlier during normal ontogeny as compared to cellular ones[344]. Indeed, in rodents, B-lineage precursors are already present in the fetal liver by day 11 of gestation, whereas low numbers of thymus-derived lymphocyte subpopulations are first identified on gestational day 17. Yet, the development of the immune system in vertebrates extends well beyond intrauterine life. Indeed, if primary organs of the immune system are progressively set up during gestation, the neonate, however, primarily relies on its innate immune system for facing infections, as well as on the passive maternal immunity ensured by the transfer of protective factors from maternal breast milk, among which is secretory immunoglobulin (Ig)

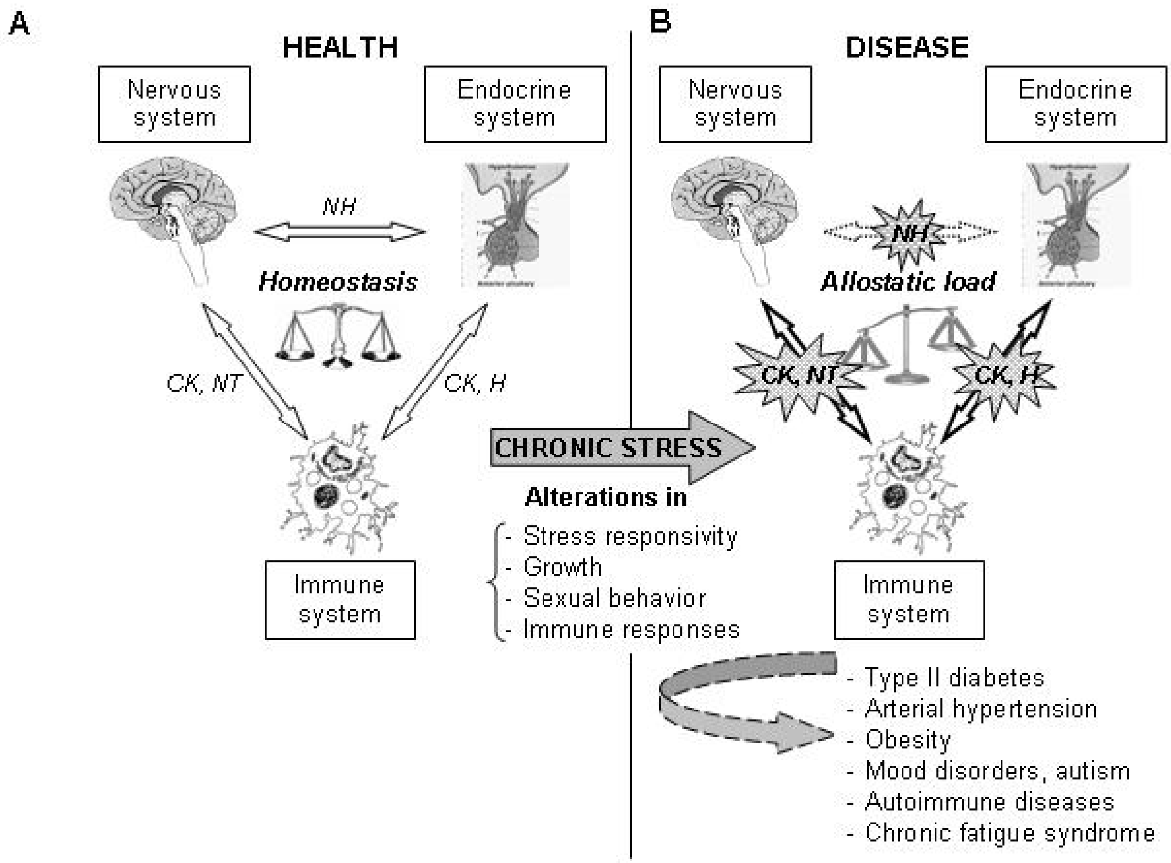

**FIGURE 3**. Cross-talk between nervous, immune, and endocrine systems in health and in disease. (A) In physiological conditions, a permanent communication between nervous, immune, and endocrine systems underlies homeostasis in vertebrates. In particular, the HPA axis and the autonomic nervous system tightly interact with the immune system to generate adaptive responses to acute stress. The constant adjustments of these three fundamental systems to the many changes of the environment are responsible for a dynamic equilibrium, ensuring the survival of individuals in almost every environmental situation. Such reciprocal influences of the central nervous system, the immune system, and the endocrine system on each other are mainly mediated by neurotransmitters (NT), cytokines (CK), neurohormones (NH), and/or hormones (H). (B) When the living organism faces chronic stress situations, lasting perturbation of homeostasis might ultimately lead to diseases. Indeed, an inadequate communication between neuroendocrine and immune systems can contribute to the physiopathology of disorders associated with immune alterations, such as the chronic fatigue syndrome or autoimmune diseases. In such conditions of maladaptation, stress triggers lasting alterations in levels of NH, NT, CK, or H. The subsequent disequilibrium of the organism’s homeostatis leads to dysfunctions in stress responses, growth, sexual behavior, and/or immune responses, the first step in the programming of more severe disorders like type II diabetes, arterial hypertension, or obesity. In extreme situations, where the organism does not succeed in returning to homeostasis, such perturbations might ultimately cause the death of individuals.

A[224]. This passive maternal transfer thus bridges the transition from an initially limited innate protection to a more complex adaptive immunity. While immunity is often considered to be a more autonomous and intrinsically controlled system, many lymphocyte responses are still quite immature at birth and require environmental priming. Indeed, the neonatal adaptive immune system, relatively naive to foreign antigens, requires the exposure to many germs during the first months/years of life in order to become refined and programmed, to develop efficiently, and to provide the organism with an adequate immunological response in face of infections. In this view, a decreased exposure to systemic childhood infections has been suggested as a major contributory factor to the subsequent occurrence of allergies and autoimmune diseases[276,315]. Although stress hormones like GC are known to exert profound effects over the development and function of the immune system[209,218], little is known about adverse early experiences on the offspring’s immunity and vulnerability to disease. It is generally acknowledged that acute stress at adulthood enhances, whereas chronic stress suppresses, the immune function[77,78,213]. In this view, considering the permanent cross-talk between nervous, immune, and endocrine systems that is initiated during fetal life, one can postulate that PS might alter the developing immune system and subsequently affect the offspring's immune function.

As already mentioned, due to the difficulties in conducting studies on human cohorts, the general consequences of PS have barely been evaluated in human beings and even fewer studies investigated the potential impact of gestational stress on the immune function in human newborns, although several reports assessed such consequences in the maternal organism. In particular, psychosocial stress during pregnancy has been shown to increase maternal inflammatory markers, such as C-reactive protein, and to alter the profile of maternal cytokines, like interleukin (IL)-6 and IL-10, towards overexpression of proinflammatory cytokines across pregnancy[61,62] (Fig. 2). These changes have been associated with the occurrence of preeclampsia, a toxic condition occurring in pregnancy and characterized by hypertension, fluid retention, and albuminuria, as well as premature labor[254]. It is thus conceivable that such dramatic modifications might threaten the immediate survival of the developing organism and/or program alterations of the immune function of the unborn child. Indeed, Kavelaars and coworkers documented an enhanced NK cell activity and a decreased T-cell proliferation in the cord blood of neonates born from mothers treated with the synthetic GC betamethasone[154], which confirmed earlier reports of functional immune deficits in preterm newborns after a maternal steroid treatment[42,178]. Moreover, several studies have suggested a link between maternal stress and T-cell differentiation of the developing immune system underlying a possible role of gestational stress in the development of asthma and atopy in genetically predisposed children[349]. Taken together, these data converge in suggesting a detrimental role of maternal stress in humans on the development of the immune competence of the unborn child.

Despite the paucity of the available data in human beings, various animal models were used to investigate the consequences of maternal stress on the development of an immune response in the offspring. Yet, data presented in the literature are often contradictory and results obtained so far greatly depend on the animal species, the nature of the stressor, the duration of stress (acute vs. chronic), the intensity and persistence of the stressor (intermittent vs. sustained), as well as the immune compartment investigated (blood vs. thymus, spleen, or lymph nodes), and the age at which the offspring is examined (neonates vs. juveniles or adults). In this view, several studies reported immunosuppressive consequences of diverse PS procedures. For example, daily restraint of gestating sows during the last 5 gestational weeks exerts an immunosuppressive effect in the early postnatal life of the offspring, characterized by a significant decrease in serum IgG and a reduced proliferative response of blood lymphocytes to mitogens[330]. Likewise, in a series of experiments using several gestational stress paradigms in rhesus monkeys, Coe and collaborators demonstrated that PS resulted in altered immune responses in the juvenile offspring, with a pronounced suppressive effect on cell-mediated immunity evidenced by a decreased proliferation of lymphocytes and a reduced secretion of proinflammatory cytokines tumor necrosis factor (TNF)-$\alpha$ and IL-6[52,54,55]. Similarly, prenatal social stress in rats decreased the total number of leukocytes in the adult male progeny, particularly of the $CD4^+$ T-helper subset, and reduced lymphocyte proliferation to pokeweed mitogen[120]. Conversely, immunoenhancing effects of PS have also been documented in the literature. For example, restraint stress in the last third of gestation in the rat led to a marginal decrease in NK cell activity in the juvenile male offspring and to an increase in NK cell toxicity in both female and male adult offspring[163]. Similarly, antenatal intramuscular injections of betamethasone in gestating ewes suppressed the endotoxin-induced inflammation in the offspring 1 day after preterm delivery, but later increased alveolar neutrophils and proinflammatory cytokine mRNA expression[152]. Furthermore, maternal disturbances early in gestation increased the proliferation of mononuclear cells from neonate rhesus monkeys in response to any stimulatory cell in the mixed lymphocyte response, whereas it was decreased in the offspring from dams that were stressed later in gestation[53]. Taken together, these results highlight the importance of both the timing in the PS paradigm and the age of the offspring in which the immune function is investigated, since opposite results may be obtained in the same animals. Finally, some authors observed no effects of PS on the humoral immune response of neonate and juvenile rats[13], or on the host defense system of the respiratory tract of young adult rats[235], whereas others found both immunosuppression and increased production of the proinflammatory cytokine IL-1β in the spleen and in the frontal cortex of juvenile rats[177]. Therefore, even if PS convincingly alters cellular and humoral immunity in laboratory animals, thus providing a focus for investigating stress-induced immune compromise, whether these effects are anti- or proinflammatory is currently a matter of debate.

Moreover, it has to be noted that most studies in the field mainly focused on short-term immune consequences of PS, either analyzing the immune system of the offspring immediately after birth, when it is not yet totally mature[207,309], or early in the postnatal life, during lactation[12,13,309], at weaning[13,309], or in the adolescence up to early adulthood[13,156,192,309,325]. To our knowledge, none of these reports described long-term effects of PS on the immune function later in adult life. In this view, we explored long-lasting consequences of PS on the immune competence of adult male rats and reported the development of a PS-evoked proinflammatory condition as rats reach full maturity[342]. In particular, PS increased the activity of the cellular immune system in rats at 6 months of age, evidenced by an increase in percentages of blood $CD8^+$ and NK cells, and a higher proliferation in response to phytohemagglutinin-A *in vitro* paralleled by an increased secretion of gamma interferon (IFN-γ). Interestingly, these alterations were undetectable in younger PS rats (7 weeks old), except for a slight increase in the mRNA expression of several proinflammatory cytokines in peripheral blood mononuclear cells. Moreover, *in vivo* neutralization of IFN-γ in young rats induced a marginal increase in IgG1, but no effects on the percentage of all circulating lymphocyte subpopulations. Finally, our work indicates that such proinflammatory alterations of the basal immune surveillance develop over time as animals grow old. The functional significance of these findings (beneficial vs. detrimental) for adult and aging PS individuals towards further insults occurring later in life is still questionable; however, it appears from this report and others that the immune system of control and PS rats would not equally face infections. Considering that IFN-γ is one of the major cytokines released in the course of T helper (Th) 1–mediated immune responses that are especially recruited for facing intracellular infections[319,328], one can speculate that PS animals could be more efficient in fighting such infections; however, if confronted with conditions requiring them to mount a Th2-orientated immune response, such as a parasitic threat or allergies[57,372], these PS organisms would need more time as compared to controls to reorientate their immune system and thus such a proinflammatory profile would be damaging for the immediate survival of these individuals. In this view, neonatal maternal deprivation in rats has indeed been shown to facilitate primary infection by nematodes and to enhance gastrointestinal inflammatory responses in the adult offspring[18]. Furthermore, considering Th1-mediated immune responses, one could even fear that the proinflammatory immune profile observed at adulthood in PS rats could later favor the development of autoimmune conditions in aging PS rats. In this view, it has been shown for example that IFN-γ–deficient mice are protected against autoimmune disease of hair follicles[98]. Thus, whatever the species considered and the PS paradigm used, data from the literature clearly indicate that perturbations of the fetal development programs are both short- and long-term modifications of the immune function. In particular, PS might grant an immediate protection towards specific environmental threats, but at the expense of an increased sensitivity to autoimmunity in the long term.

## Putative Mechanisms Underlying Prenatal Stress-Evoked Immune Alterations

While examining the mechanisms underlying these reported PS-evoked alterations of immune functions in the offspring, one has to consider two hypotheses. First, as already demonstrated for other parameters of the developing organism, like for the glucose transport (see, “Role of Nutrients in Growth” above), one could suspect a defect in the feto-placental barrier and, more especially, a deficit in the passive transfer of the maternal immunity to the unborn child and/or to the neonate. However, in mice, the transplacental transfer of total and herpes simplex virus–specific IgG antibodies, and the subsequent protection of neonates towards herpes simplex virus infections, has been shown to persist despite acute maternal stress and the resulting increase in GC both in maternal and fetal organisms[375]. Similarly, the prenatal transfer of IgG in rhesus monkeys has been documented to resist both a DEX treatment and a prolonged period of stress during pregnancy[51]. Both studies thus suggest that transplacental immunity is resilient to maternal stress, at least in rodents and primates. Moreover, if such a mechanism would trigger alterations of the immune system in PS descendants, these dysfunctions would then last until weaning, while the neonate primarily relies on the passive maternal immunity for its own immune protection. In this case, one would expect a partial (at least, if not full) recovery of the immune function in adolescent and adult individuals. In this view, such a deficit in the passive transfer of maternal immunity would not provide an explanation for the long-lasting consequences of PS on the immune function and appears not to be the main cause of the documented PS-induced alterations of immunity, thus suggesting that PS-evoked alterations in the *in utero* development of immune organs might rather be involved.

Indeed, most studies focused on GC for explaining the mechanisms by which gestational stress can have lasting consequences on the developing organism. As far as the immune system is concerned, stress at adulthood is known to influence the thymic output of $CD4^+$ and $CD8^+$ cells[379] on which depends the peripheral $CD4^+/CD8^+$ ratio[68]. In this view, since (1) altered circulating GC levels have been reported in PS fetuses in several PS models[32,85,185,234,322], (2) GG modulate the surface expression of CD4 and CD8 markers[369], and (3) the thymic selection of T cells is directly influenced by the level of CD4 or CD8 expression[97,275], thus altered levels of GC occurring in PS fetuses might likely affect T cell selection and thereby the peripheral $CD4^+/CD8^+$ ratio as reported in many studies[12,177,192,342]. However, besides the highly probable role of GC in programming dysfunctions of the immune system in PS progenies, one cannot exclude the involvement of other stress-related molecules, such as catecholamines. Indeed, in rodents, for example, it is well known that the sympatho-adrenal system of PS animals exhibits a greater sensitivity to stress at adulthood[364]. Since the thymus is innervated by noradrenergic fibers[83], PS might also alter the noradrenergic innervation of the developing thymus, thus affecting thymocyte selection and leading to an altered $CD4^+/CD8^+$ ratio as documented in several studies[12,177,192,342]. Considering that immune cells express receptors for both hormones and neurotransmitters[280], we suggest that the altered peripheral $CD4^+/CD8^+$ ratio in young and adult PS progenies probably results from the combination of both increased circulating GC and impaired noradrenergic innervation in the fetal and neonatal developing organism. Moreover, we and others have shown that maternal perturbations do not stop at birth with the cessation of the stress protocol, but continue throughout lactation[69,72,307]. In most experimental paradigms, PS animals are raised by their biological mothers, which emphasizes a further potential impact of GC and other stress-related factors on thymocyte selection during the neonatal period[341], and further underlines the potential consequences of a disturbed maternal care on the setup of the immune system during the first months/years of life. In this view, one could expect a reversion of such PS-induced alterations of immune parameters by cross-fostering experiments as already demonstrated for other systems such as the HPA axis reactivity at adulthood[14,19,20,202]. Finally, one has to keep in mind that PS-evoked alterations of the immune function might further enhance the dysfunctions of other physiological systems, such as the HPA axis. In this view, mice exposed to corticosterone during the last week of gestation and until weaning display an increased expression of the mRNA coding for the leukemia inhibitory factor (LIF) in the hypothalamus as well as altered emotional behaviors[243]. Since the proinflammatory cytokine LIF is known to stimulate ACTH secretion in response to emotional and inflammatory stresses[8,48], and has been recently linked to depressive-like behaviors[244], one can thus expect that the up-regulation of the central LIF mRNA expression might further alter the reactivity of the HPA axis in PS progenies as well as their behavioral responses in the face of stressful situations. These results further underline the permanent cross-talk between nervous, immune, and endocrine systems that is highly susceptible to homeostasis perturbations.

In conclusion, taken together, these observations indicate that events of the fetal life can persistently influence the function of the immune system of individuals long after birth and tilt the balance away from health toward illness. In mammalian species that are quite totally dependent on the mother for survival, it can also make the neonate particularly vulnerable to subsequent disturbances of the maternal care, which is particularly critical in the setting up of immune functions. One has to remember, however, that the primary impact of gestational stress on the progeny aims at favoring their immediate survival as well as that of the maternal organism. In this view, sometimes the prenatal disturbance is just salient enough to shift the regulatory set points of the physiological systems that will mature later after birth, such as the adaptive immune response. But in other cases of more severe gestational perturbations, the impact is so potent that the trajectory of fetal development is derailed towards pathology. Our research in rodents indicates that chronic maternal stress during gestation has this type of saliency, capable of leaving a lasting mark on behavior and physiology that can persist into adulthood, and eventually program lasting immune dysfunctions that might render individuals more susceptible to autoimmunity. Thus, such studies further support the hypothesis that pathologies of the adulthood, including those of the immune system, can be programmed during prenatal/perinatal life as previously suggested by Barker[17] and others.

## PRENATAL STRESS AND THE PROGRAMMING OF DISEASES OF THE ADULT

The role of PS in the programming of adult diseases has gained importance over the past decades since the initial work of Barker[17]. Indeed, a limited supply of nutrients during the course of fetal development has been associated with the occurrence of coronary heart diseases and related disorders in adults. For obvious ethical and methodological concerns, animal models of gestational stress have been widely used for deciphering the mechanisms of PS-programmed diseases of the adult[223]. Notwithstanding the role of PS in the development of mood-related and metabolic diseases, the evolutionary role of all modifications observed in the fetuses of stressed mothers must be underlined. Up to now, PS has mostly been considered as deleterious for the survival of individuals. The question needs to be discussed in terms of adaptation in a truly unfavorable environment from an ecological perspective. In fact, most studies conducted on PS animal models have been performed within strict and controlled laboratory conditions that are usually distant from natural living conditions of the species considered. Moreover, data obtained from human cohorts are often inconsistent, as previously mentioned. As a consequence, one has to keep in mind these restrictions during the present discussion.

### Prenatal Stress and the Occurrence of Mood-Related Disorders

Among PS-induced disturbances, modifications of emotional behavior are often described in various species, including humans[365,366]. In fact, in most animal models, numerous alterations reported in PS offspring are highly similar to human symptoms of anxiety and depression. As an example, adult PS rats exhibit alterations of circadian rhythms and a higher vulnerability to drugs that are classically associated with increased anxiety or depression-like behaviors[75,203,216,366] (see also “PS and the Programming of the HPA Axis” above). Similarly, behaviors classically disrupted in schizophrenic patients, like the sensorimotor gating reflected by the prepulse inhibition and the sensory gating reflected in measures of the N40 auditory evoked potential, have been described in rodent progenies whose mothers were exposed to infection or to a variable stress paradigm[164,212]. In humans, a tight connection between early stress exposure and the occurrence of anxiety disorders during adolescence has been evidenced[251]. The Avon Longitudinal Study of Parents and Children revealed that mood disturbances in pregnancy, anxiety and/or depression, are associated with altered salivary cortisol levels in 10-year-old children[230] and result in lasting disturbances of sleep in toddlers, like nighttime waking, nightmares, or difficulties in falling asleep[231]. In another cohort, the Dutch Hunger Winter Study, the sharply defined period of famine has been associated with an increased risk of hospital treatment for major affective disorders[30]. Moreover, this study provides accurate data on the impact of middle-to-late gestational nutritional deficiency in the etiology of major affective disorders, like unipolar or bipolar depression, which affects both men and women, although the effects appear to be somewhat weaker in the latter. These data further underline the high prevalence of the risk of schizophrenia and of schizoid/schizotypal personality disorder in the birth cohort exposed to a severe famine during early gestation as initially showed by Susser and coworkers[317,318]. Such a disorder has been associated with increased brain abnormalities, predominantly white matter hyperintensities[145]. Altogether, these results highlight the possible existence of a continuum of psychiatric diseases, from affective disorders to schizophrenia, whose type and severity may be related to the gestational timing when famine occurs. In addition, a rising incidence in autism has been noticed since the early 1980s that cannot be explained only by the change in diagnostic criteria[262]. Although this neurodevelopmental disorder is considered to be one of the most genetically determined, industrialization of our modern societies has triggered dramatic modifications in the prenatal environment that have been hypothesized to account also for the increased occurrence of this psychiatric disease[262]. Among factors implicated in the etiology of autism, the hyperdopaminergic state is commonly pointed out[1,273]. In this view, chronically elevated maternal dopamine through diet, exposure to toxins like mercury (vaccines, dental amalgams, etc.), medications, illness/fever, or psychosocial stress can significantly heighten the risk for autism[262]. Beyond the associated impact of pollution and toxins, one may underline the role of diet since, as mentioned earlier, it has been crucial in the hominid evolution. We currently eat more meat and more proteins that provide larger amounts of amino acids like tyrosine. This increased supply in tyrosine and/or phenylalanine to the brain can thus augment the synthesis of dopamine. Hence, the increased rate of autism in our industrialized societies might be not only due to genetic deficits or a better DMS IV diagnosis, but to combined factors chronically affecting the mother during pregnancy.

In conclusion, both animal models and studies conducted on human cohorts clearly suggest that PS may be at the origin of neurodevelopmental modifications that later lead to amended emotional behavior, such adverse effects being mediated by alterations of the early neuroendocrine programming. These behavioral responses were probably adapted to a formely hostile environment where a higher vigilance, an augmented response to stress, and/or an increased anxiety would have favored the immediate survival of individuals. However, nowadays, such behaviors appear to be inadequate in our urbanized societies where, in the absence of predators, the "*struggle for life*" does not exist anymore with the same physical intensity. The resulting deficiencies may alter social integration of such PS individuals and eventually lead to the development of affective disorders that can give rise to more severe psychopathologies.

## Prenatal Stress and the Occurrence of Metabolic Disorders

Substantial evidences emphasize the association between a too-small birth weight, an index of poor fetal growth, and/or preterm delivery, with elevated risks of developing metabolic disorders at adulthood[16,17,304]. In this view, the metabolic syndrome, characterized by the occurrence of glucose intolerance associated with impaired insulin action or insulin resistance, hypertriglyceridemia, and hypertension has been related to a small birth weight[250]. These diseases have also been associated with chronically elevated levels of plasma GC[288].

### *Type II Diabetes*

In rodents, an association between a low birth weight and the subsequent development of type II diabetes has been shown in the adult offspring generated from mothers exposed to food restriction or emotional stress, like restraint during gestation[107,127,128,185]. In particular, Lesage and coworkers[185] demonstrated that PS rats aged 24 months exhibit hyperglycemia under basal conditions and after a glucose load, but no variation in insulinemia. These changes reflect the occurrence of type II diabetes. Further evidences of a determining role of the fetal environment in the susceptibility towards type II diabetes arose from an epidemiological study among men in their 60s[125]. Men who displayed both lower birth weights and lower weights at 1 year of age were more likely to develop poor glucose tolerance and type II diabetes at adulthood. In support of these data, twin studies have demonstrated discordances for type II diabetes with the diabetic twin having a significantly lower birth weight than his nondiabetic sibling regardless of zygosity[259]. The causes of this metabolic disease remain incompletely elucidated, however. Besides the data highlighting the influence of the fetal environment on the occurrence of diabetes in old age, Hattersley and Tooke[130] proposed the "*Fetal Insulin Hypothesis*", which associates both impaired fetal growth and susceptibility to type II diabetes at adulthood with genetically determined insulin resistance. However, this alternative hypothesis has been criticized, for no association with birth weight has been documented in cases of polymorphisms known to be associated with insulin resistance[84]. Consequently, even if genetic polymorphisms may have an impact in the development of diabetes, the impaired fetal growth caused by an altered gestational environment has stronger influences on the apparition of the disease. In this view, future research should assess the relative contributions of genetic factors and the fetal environment in the occurrence of low-birth-weight offspring.

### Obesity

Like for type II diabetes, a small birth weight has also been associated with another component of the metabolic syndrome, namely the central obesity[250]. However, the occurrence of this disease certainly hinges on the intrauterine development as well as on postnatal influences. In fact, biological features of obesity like hyperphagia, increased plasma leptin, hyperglycemia, or adipocyte hypertrophy are associated with a rapid neonatal catch-up growth after maternal nutrient restriction during gestation[24,76]. On the contrary, maternal food restriction during lactation triggers a delayed catch-up growth, restoring biological features to control levels with normal body fat and plasma leptin. Thus, the programming of obesity and related disorders in the offspring may depend on the maternal environment as well as on the timing of nutrient reduction and/or nutrient availability throughout the perinatal development. Moreover, as stressed out by Prentice[261], the early programming of the "*fat-brain axis*" is not comparable between humans and rodents. Indeed, the appetite regulatory system and the feedback loops from adipose tissues seem to be set up prenatally in humans, but not in rodents. Therefore, except for a study showing a higher obesity among young men whose mothers endured the Dutch winter famine during the first and second trimester of pregnancy[268], the link between prenatal undernutrition and the further development of obesity at adulthood is tenuous and inconsistent. Conversely, more consistent data gathered in humans suggest a predisposition to hyperphagia and obesity in relation to a higher maternal weight gain during pregnancy and/or an early postnatal overfeeding[261]. Finally, one can also mention an anecdotic link between maternal diet and PS consequences on infant temperament[267]. Indeed, mothers who reported consuming chocolate daily rated their infants at 6 months of age as more positively reactive and active. Moreover, while PS predicted more negatively tuned maternal ratings of the infant temperament, this effect was not observed in stressed mothers who reported weekly or daily chocolate consumption. These data are among the first to suggest a positive impact of a particular kind of food to reverse some PS negative effects on the progeny.

### Cardiovascular Diseases

Another component of metabolic diseases also linked to a small birth weight is arterial hypertension. In humans, increases in both diastolic and systolic blood pressure and heart rate have been described following a mild psychological stressor in women who were smaller at birth, but not in men, suggesting an *in utero* programming of cardiovascular reactivity[351]. Moreover, a rapid postnatal growth has been described to diminish the longevity. In rats, the life span is adversely affected when animals exhibit a rapid catch-up growth following *in utero* growth restriction; in humans, similar effects of the catch-up growth are observed with adverse long-term outcomes in terms of blood pressure or death from cardiovascular diseases[127]. In fact, maternal hypoxia, malnutrition, or restraint stress lead to offspring with similar cardiovascular responses to acute stress at adulthood and no basal differences in hemodynamics[147,249,327]. In particular, adult PS animals exposed to acute mild stress present with an increased systolic blood pressure with an extended recovery delay. In general, PS female offspring display a greater and more prolonged cardiovascular response than males[147]. This impaired responsiveness to an adverse environment could be deleterious in the long term, especially in the case of chronic stress leading to arterial hypertension. High levels of maternal GC are thought to later program cardiovascular diseases. Indeed, GC are known to increase blood pressure in adult animals[337].

Similarly, patients with Cushings syndrome (chronic overproduction of cortisol) are frequently hypertensive[368]. Different experiments were carried out to demonstrate the role of elevated maternal GC on the development of hypertension in the offspring. In particular, the treatment of undernourished (low protein diets) gestating rats with metapyrone (an inhibitor of 11β-hydroxylase) inhibits the corticosterone synthesis in both the dam and fetuses, thus preventing the development of hypertension in the adult PS offspring[172]. The effects of an excess maternal GC might be direct or indirect, acting, for example, at the hypothalamic level (AVP neurons of the PVN) or at the level of vascular smooth muscle cells, like thoracic aorta, where binding of GC on type II receptor augments artery resistance, thus eliciting increased blood pressure[171,292]. Moreover, GC can also modify the development and maturation of specific organs involved in the control and maintenance of blood pressure[295]. In this view, a low maternal weight at the end of pregnancy and a low birth weight have been associated with a decreased arterial compliance capacity associated with a reduced vessel size in individuals born as term singletons around the period of the Dutch famine whereas no change on the carotid size or stiffness has been observed in the descendants[240].

Since neuroendocrine and physiological modifications induced by the deleterious maternal environment may give PS offspring the ability to survive in a poor milieu, i.e., where a catch-up growth is impossible, the above-reported data account for an indirect support of the evolutionary significance of PS alterations. In this view, one may speculate that an immediate prevention of the rapid neonatal catch-up growth in PS infants may reduce the risk of adult-onset obesity and its related disorders, like hypertension and type II diabetes.

## Is Prenatal Stress Programming of Adult Diseases Mandatory?

As mentioned in the previous sections, the severity of the disorders displayed by the PS offspring is a function of the developmental window at which the maternal stress occurs. Nevertheless, several data obtained in both rodents and humans point out the importance of the postnatal environment on the offspring development. In rats, the role of early environmental events, like infantile stimulation (or handling), has been clearly established in the optimal development of the progeny[186,310,326,363]. Indeed, repeated short-duration handlings of pups appear to exert a positive long-term effect on their HPA axis reactivity when faced with stressors in comparison with nonhandled animals[187,335]. It has been suggested that the effects of handling are mediated by changes in maternal care, like increases in the licking/grooming of handled pups by the mother[191]. In fact, newborn rodents, virtually immobile and incapable of body temperature maintenance, are strongly dependent on the initiation of a specific set of maternal behaviors for their survival that include, for example, sniffing and exploration of pups, mouthing, pup retrieval, and nursing[264]. Moreover, a direct relationship between maternal care and postnatal individual differences in behavioral and HPA axis responses to stress has been established[93,96,104]. In a restraint PS rat model, early adoption of the offspring, irrespective of the stress experience of the foster mother, reverses the effects of PS; adoption *per se* increases maternal behavior and decreases the HPA axis activity in the adult offspring following acute stress[14,70,202]. In mammals, the maternal behavior, whose initiation and maintenance involve specific neural circuits controlled by various genetic factors, is a highly conserved set of behavioral capacities that are crucial for the reproductive success[181]. Variations in maternal, and more generally parental, care early in the life of the offspring might durably influence their development and vulnerability to illness throughout life[37,151]. In a rat model of high maternal care (high vs. low licking/arched-back nursing dams, LG-ABN), the offspring exhibit differences in the behavior (low emotionality), neurobiology (high levels of GABAa receptor in amygdala and locus coeruleus), and neuroendocrine responses to stress (reduced plasma ACTH and corticosterone, enhanced GC feedback) in comparison with offspring from low maternal care dams[35,36,44,45,92,93,94,95]. These data support the hypothesis of a programming of the offspring's neuroendocrine and behavioral responses to stress mediated by the behavior of the mother. Furthermore, such a maternal influence on the programming of the progeny has been reinforced by recent data showing that offspring from high-LG-ABN mothers exhibited differences in DNA methylation, as compared to offspring from low-LG-ABN mothers[359]. These effects emerge over the first week of life, persist into adulthood, are reversed with cross-fostering, and are associated with altered histone acetylation and transcription factor binding to the GC receptor promoter. DNA methylation thus alters GC receptor expression through modifications of the chromatin structure[211,359,360,361,362]. In line with these arguments, Cameron and coworkers[37] suggested the existence of a phenotypic plasticity associated, for example, with parental investment that may be greater in more complex species like mammals. However, the influence of the maternal exposure to adverse environments on the mother-offspring interaction appears to be a common feature across all species caring for their young. In such a situation of adversity, the development of defensive responses to threats and/or of reproductive strategies might be altered in the progeny. Furthermore, epidemiological studies suggest that environmental adversity can alter parental care and thus influence child development. Similarly, in rodents, restraint stress during gestation exerts lasting effects on emotional reactivity of the dams, characterized by a higher anxiety and less care to the offspring[69]. In another model of PS in rodents, gestational stress can directly alter maternal care *via* the neuroendocrine systems that normally regulate this behavior[45]. These data also stress a nongenomic transmission mechanism of the effects of environmental adversity on maternal care across generations. Taken together, these studies emphasize the critical influence of parental care, especially after birth when children or pups develop or achieve their development. Deficits in parental attention combined to PS may have deeper effects on the offspring's development, resulting in emotional and/or cognitive anomalies. As suggested by Hinde[137], natural selection has shaped the offspring to subtle changes in parental behaviors as a forecast of the environmental conditions they will face following separation from the parents. Besides the importance of parental care, an enriched environment at birth and until weaning can also reverse PS deleterious consequences by restoring abnormal behaviors like emotional reactivity or motor skills[46]. In fact, rat offspring reared in an enriched environment display increased social and play behaviors, a reduced emotionality, a reversion of several immunological alterations, and a decrease in the enhanced morphine-induced place preference[177,214,374]. Moreover, a longitudinal cross-housing study reveals that the postnatal environment could attenuate the effects of the prenatal condition on cognitive functions and more especially on synaptic structural changes in the hippocampus, with a higher immunoreactivity of neural cell adhesion molecule (NCAM), BDNF, and synaptic-vesicle protein (SYP), markers of neuronal and/or synapses development[166]. Indeed, a small hippocampal volume has been associated with both low birth weight and low maternal care in human females[34]. Thus, the detrimental effects of PS can be counteracted by an enriched stimulating environment after birth. The beneficial effect of enriched environment on brain development and behavior is corroborated by data from an animal model of autism induced by prenatal exposure to valproic acid. Specific environmental experiences, such as behavioral-cognitive therapy, might then attenuate autistic features and favor damage rehabilitation[188,290].

In conclusion, the gestational and early postnatal maternal programming appears to have contributed to the adaptation and survival of species in ever-changing environments. Such developmental responses have been conserved and have likely contributed to the epidemic occurrence of the metabolic syndrome, and the development of various mood disorders or psychiatric diseases. Several questions remain in abeyance, however, for a clear understanding of the mechanisms that can regulate and/or modify this maternal programming. In the developmental environmental context, such a fetal and/or neonatal plasticity might be used as windows of opportunity to prevent potential detrimental effects of maternal programming and/or enhance its beneficial impacts.

## GENERAL CONCLUSION AND IMPLICATIONS FOR HUMAN BEINGS

In the present review, we have stressed the idea according to which an immature organism relies quite heavily on its environment, namely the maternal womb, from which it gathers information that will guide its harmonious growth and development. In the gestational context, this would imply an early programming of several physiological/biological systems to ensure the fetal-maternal interaction (Fig. 4). In particular, the nervous system, together with its two other counterparts, the endocrine and the immune systems, appears to be intrinsically programmed from very early on to react in a responsive manner and to learn from environmental stimuli in order to adjust internal parameters of the developing embryo/fetus to its environment, i.e., to the maternal one. In this view, such an early programming aims at maintaining the fetal homeostasis. This permanent responsiveness to the context allows for a greater flexibility in the developmental trajectory and thus seems to have been selected through evolution for ensuring the survival both at the level of the individual and at the level of the species. However, there is a potential cost to this apparently endless flexibility: an increased risk for an adverse outcome if the stimulation exceeds the

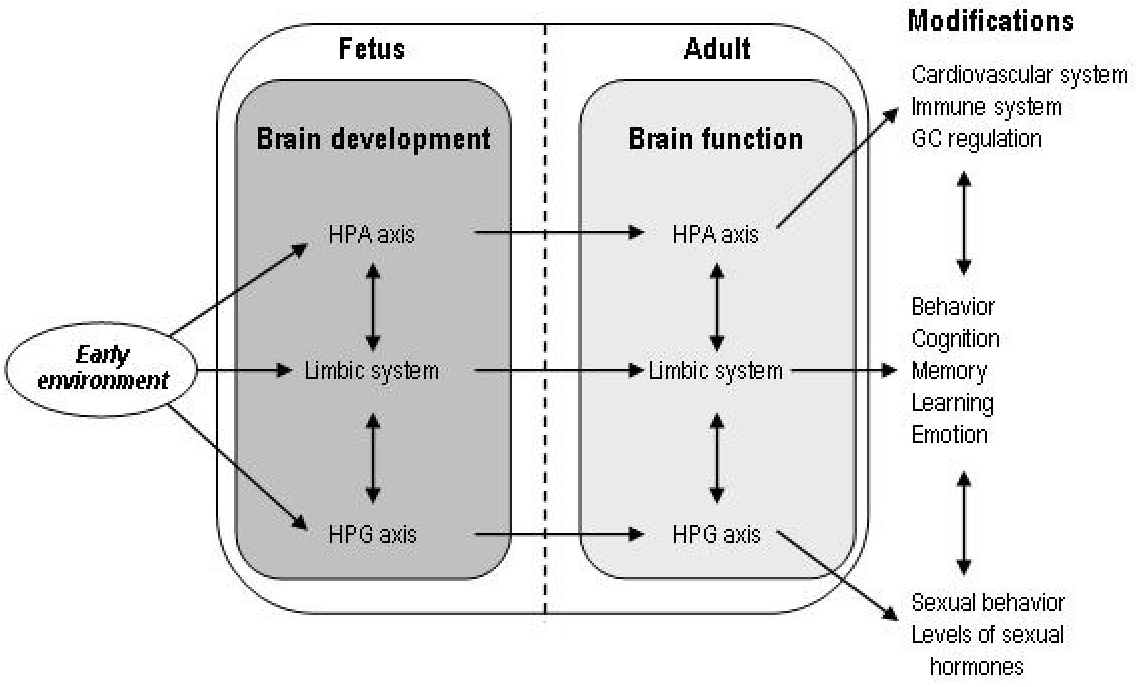

**FIGURE 4.** Developmental environment and early programming. Environmental influences are often considered to begin after birth, however, the prenatal *in utero* environment plays also a fundamental role in the early programming of the physiological integrity of the fetus. Ever-changing environmental conditions can indeed produce different outcomes on the development of the organism by impacting the fetal brain. In particular, early stressful events during gestation might shape the developing brain, notably the HPA and HPG axis, as well as their common interface, namely the limbic system. Thus, any event occurring during the perinatal period may affect various aspects of the neuroendocrine programming. Such early environmental changes can be either advantageous and promote an optimal development, or deleterious and alter the development, subsequently programming modifications of cardiovascular system, immune function, GC regulation, behavior, cognition, memory, learning, emotion, sexual behavior, or levels of sexual hormones, etc. and ultimately leading to the development of adult diseases as initially suggested by Barker[17]. GC: glucocorticoids; HPA : hypothalamo-pituitary-adrenal axis; HPG : hypothalamo-pituitary-gonadotropic axis tolerable limits of the developing organisms. Nevertheless, this perspective should not mean that all environmental changes are to be considered as deleterious for the fetus. Indeed, sometimes the disturbance will be just salient enough to shift the regulatory set points of the physiological systems that will mature later after birth. However, in other cases, the challenge is so potent that the trajectory of fetal development is deviated towards pathology. In particular, numerous studies including our own indicate that extended periods of maternal stress during gestation, and exposure of the fetus to alcohol or drugs like DEX, are capable of leaving a lasting mark on behavior and physiology that can persist into adulthood.

Moreover, one has also to keep in mind that most information gathered in the field of perinatal programming emerged from animal-based studies. With regard to the relevance of these findings for our species, we would like to suggest that human babies are somewhat more resilient to PS adverse consequences, in part because of a greater reliance on the more protracted period of postnatal growth. In this view, lactating rats (postnatal day 13) and monkey infants are born with a brain, respectively, 52 and 60% of the adult size, whereas the human neonate by comparison has a brain only 24% of the adult volume[56,110]. Thus, a prenatal insult in rodents or monkeys is likely to have considerably more developmental impact with less chance for postnatal recovery since the potential for neonatal plasticity within the brain is more limited. However, the developing human central nervous system may be more vulnerable to environmental perturbations than any other system. Indeed, it develops over a much longer period of time (~18–20 years) as compared to other species, it has limited repair capacities, and the programming of neurotransmitter systems during critical developmental periods might affect the organism’s response to any subsequent experiences. Despite the significant and persistent effects of PS on the neuroendocrine programming documented in the literature, most of these changes should be viewed as being within a tolerable range of modulation, with little adverse consequences under undisturbed and unprovoked conditions. It is only in the context of environmental challenge that one can really see the potential vulnerability of PS organisms.

## ACKNOWLEDGMENTS

The authors would like to acknowledge the warm support and critical reading of the manuscript by Drs. M.C. Chartier-Harlin and I. Wolowczuk.
